# Dysbiosis of nasal microbiota associated with hippocampal neuroimmune alterations in a mouse model of eosinophilic chronic rhinosinusitis

**DOI:** 10.3389/fimmu.2026.1811570

**Published:** 2026-09-03

**Authors:** Ryuichi Imai, Yuzuki Sugimoto, Takako Osaki, Sanae Hasegawa-Ishii

**Affiliations:** 1 Faculty of Health Sciences, Kyorin University, Tokyo, Japan; 2 Department of Preventive Medicine, Kyorin University School of Medicine, Tokyo, Japan

**Keywords:** dysbiosis, eosinophilic chronic rhinosinusitis, hippocampus, nasal microbiota, neuroimmune alterations

## Abstract

Chronic rhinosinusitis (CRS) is a chronic inflammatory disease of the nasal cavity and paranasal sinuses. Eosinophilic CRS (ECRS) is a refractory subtype characterized by persistent type 2 inflammation. Epidemiological and animal studies suggest that CRS may affect brain function and that microbiota dysbiosis may contribute to the pathophysiology of CRS. However, the relationship between microbiota alterations and neuroimmune changes remains poorly understood in CRS. In this study, we investigated nasal and gut microbiota together with glial and inflammatory gene expression in the olfactory bulb (OB) and hippocampus in a mouse model of ECRS, and examined their associations. The nasal and cecal microbiota were analyzed by 16S rRNA gene amplicon sequencing, and glial and inflammatory gene expression in the OB and hippocampus was assessed by real-time RT-PCR. Associations between microbial taxa and brain gene expression were analyzed using MaAsLin2. While alpha diversity was unchanged, beta diversity of both nasal and gut microbiota differed significantly in ECRS mice. Differential abundance analyses identified several nasal and gut bacterial taxa altered in ECRS. In parallel, expression of glial markers was increased in the OB and hippocampus, and expression of the pro-inflammatory cytokine gene *Il-6* was elevated in the hippocampus. Notably, multiple nasal microbial taxa, but not gut bacteria, were significantly associated with hippocampal glial and inflammatory gene expression. These findings indicate that ECRS is accompanied by dysbiosis of the nasal and gut microbiota together with hippocampal neuroimmune alterations and suggest a potential link between nasal microbiota composition and hippocampal neuroimmune alterations.

## Introduction

Chronic rhinosinusitis (CRS) is a chronic inflammatory disease of the nasal cavity and paranasal sinuses lasting for at least 12 weeks. CRS causes olfactory impairment due to damage to olfactory epithelium and nasal congestion, taste disorders resulting from impaired flavor perception, and sleep disturbances, all of which contribute to a reduced quality of life (1–4). Moreover, increasing evidence suggests that CRS is also associated with neuropsychiatric manifestations, including depression, anxiety, and cognitive impairment (5–8). Consistently, animal studies using models of allergic airway inflammation, such as allergic rhinitis, have reported anxiety- or depression-like behaviors in mice (9–12). These findings suggest that CRS is not merely a local peripheral inflammatory disease but may also affect central nervous system function.

Eosinophilic CRS (ECRS) is a refractory subtype of CRS characterized by frequent recurrence. It is associated with type 2 helper T cell (Th2)-dominant inflammation and marked eosinophilic infiltration in nasal polyps. Thymic stromal lymphopoietin (TSLP) is considered to be one of the key upstream cytokines promoting Th2-type inflammation, including ECRS, through activation of basophils and group 2 innate lymphoid cells (ILC2), which subsequently produce IL-4, IL-5, and IL-13 (13, 14). However, the precise etiology and pathophysiological mechanisms underlying ECRS remain unclear.

Increasing evidence suggests that dysbiosis of mucosal microbiota can exert biological effects beyond their local anatomical sites and contribute to pathological processes in remote organs. For example, gut microbiota dysbiosis has been implicated in metabolic diseases and neurological disorders through the gut-brain axis (15, 16), whereas dysbiosis of other mucosal microbiota, such as the oral microbiota, has been associated with cardiovascular and other systemic diseases (17, 18). These findings highlight the importance of mucosal microbiota as regulators of host physiology beyond the local tissue environment. Recently, increasing attention has also been directed toward the role of gut and nasal microbiota in CRS and their potential systemic effects. Several studies have reported alterations in gut microbial community composition, including significant changes in the relative abundance of specific bacterial taxa, compared with healthy controls (19–21). In addition, comparative analyses of nasal microbiota in patients with CRS, ECRS, and healthy subjects have demonstrated significant differences in microbial diversity and dominant bacterial taxa (22–24). These observations suggest that dysbiosis of the gut and nasal microbiota may contribute to the pathophysiology of CRS and influence brain function through immune, neural, and humoral pathways. However, it remains unclear whether alterations in gut and nasal microbiota are associated with neuroimmune changes in the brain. In addition, despite the increasing availability of patient-based studies, studies investigating gut and nasal microbiota in animal models of CRS remain limited.

In our previous studies, we established a mouse model of nasal inflammation by intranasal administration of lipopolysaccharide (LPS). Shortly after LPS administration, immune cells infiltrated the olfactory mucosa and secreted pro-inflammatory cytokines, inducing acute nasal inflammation (25, 26). This local inflammation subsequently triggered neuroinflammation in the olfactory bulb (OB), characterized by upregulation of inflammatory cytokines and chemokines, immune cell infiltration, and activation of microglia and astrocytes (27). Repeated intranasal LPS administration resulted in chronic nasal inflammation, leading to the loss of olfactory sensory neurons and marked atrophy of the OB (28, 29). Notably, chronic nasal inflammation also induced dysbiosis of the gut microbiota in both adult and neonatal mice (30, 31). These findings suggest that nasal inflammation can influence both neuroimmune changes and microbiota composition. However, the mechanisms linking nasal inflammation to gut microbiota dysbiosis remain unclear. Moreover, whether microbiota alterations are associated with neuroimmune changes in the brain has yet to be determined.

The hippocampus is critically involved in emotional regulation and cognitive function (32) and is anatomically and functionally connected to the olfactory system via the OB and entorhinal cortex (33). This pathway functionally links olfactory processing with hippocampal memory circuits, as demonstrated by coordinated entorhinal-hippocampal activity during odor-associated learning (34). These anatomical and functional connections make the hippocampus a relevant target for assessing the impact of nasal inflammation on neuroimmune changes.

In the present study, we hypothesized that alterations in the nasal and gut microbiota may be associated with neuroimmune changes in the OB and hippocampus, two brain regions directly or indirectly connected to the olfactory pathway. To test this hypothesis, we investigated alterations in the nasal and gut microbiota together with gene expression in the OB and hippocampus within the same mouse model of ECRS. We further statistically examined the associations between microbiota composition and brain gene expression using MaAsLin2. Although this study does not establish causal relationships between bacterial taxa and brain gene expression, it provides foundational data for exploring potential interactions among the nasal microbiota, gut microbiota, and neuroimmune alterations in ECRS. These findings may also guide future studies investigating the clinical relevance of these interactions in patients with CRS/ECRS.

## 2 Materials and methods

### 2.1 Animals and experimental design

ECRS model mice were generated according to the method described by Kagoya et al. (35), with minor modifications. Briefly, eight-week-old male C57BL/6JmsSlc mice (Sankyo lab, Tokyo, Japan) were topically treated with 2 nmol of MC903 (Tocris Bioscience, Bristol, UK) dissolved in 20 μL of ethanol, along with 100 μg of ovalbumin (OVA; Sigma-Aldrich, St. Louis, MO, USA) in 20 μL of phosphate-buffered saline (PBS), following tape stripping on the right ear for 2 weeks (6 days/week). Subsequently, the mice received intranasal administration of 20 μL of OVA (120 μg/μL) to the right nostril for an additional week (7 days). Control (CONT) mice were treated with ethanol and PBS for 2 weeks, followed by intranasal administration of PBS for 1 week.

**Figure 1:**
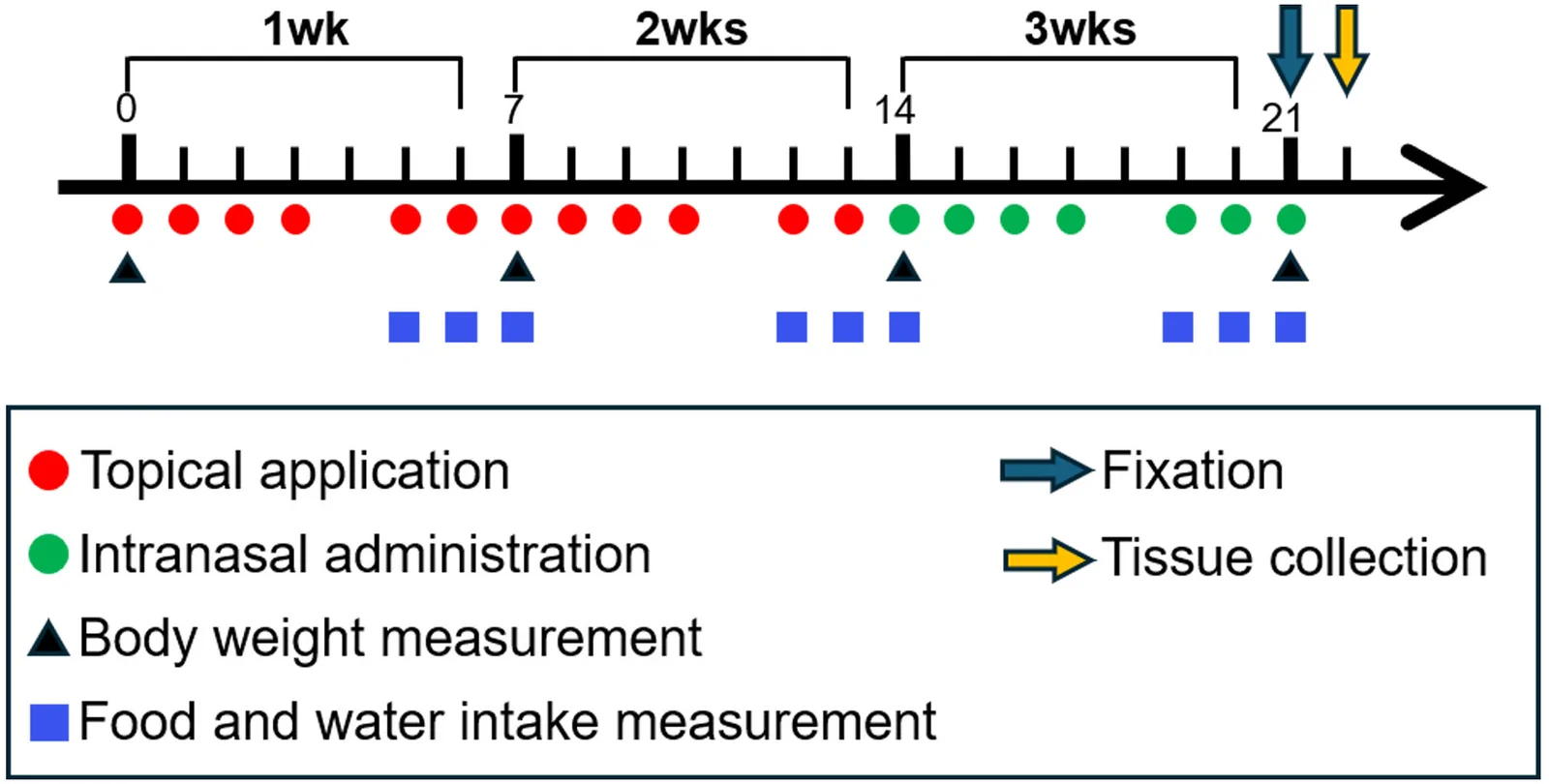
Experimental procedure. Mice in the ECRS group received topical applications (red dots) of MC903 and ovalbumin (OVA) to their right ear for 2 weeks (6 days/week), followed by intranasal administration (green dots) of OVA to their right nostril for 7 days. Control mice received topical applications of ethanol and PBS, followed by intranasal administration of PBS according to the same schedules. Body weight was measured once per week (black triangles), and food and water intake were measured for three consecutive days at the end of week 1, 2, and 3 (blue squares). A subset of mice was fixed for histological analysis (blue-green arrow). Nose and cecal contents were collected for 16S rRNA gene analysis, and fresh brain tissues were used for quantitative RT-PCR (yellow arrow).

The experimental design is summarized in **Figure 1**, and sample sizes for each experiment are provided in the corresponding Materials and Methods sections and figure legends. Independent cohorts of mice were used for different analyses; therefore, sample sizes differed among experiments. Because cecal contents could be collected before perfusion fixation, cecal samples from some animals used for histological analysis were also included in the gut microbiota analysis, resulting in a larger sample size for the gut microbiota analysis than for the nasal microbiota analysis.

All protocols were approved by and all methods were performed in accordance with the guidelines of the Institutional Animal Care and Use Committee of the Kyorin University Faculty of Health Sciences. In addition, this study was carried out in compliance with the ARRIVE guidelines.

### 2.2 Histological preparation

Mice were deeply anesthetized with ketamine (100 mg/kg body weight) and xylazine (10 mg/kg) and transcardially perfused with PBS, followed by 4% paraformaldehyde in PBS as a fixative. The heads were decapitated and immersed in the same fixative overnight at 4 °C, then decalcified in 2x K-CX solution (Falma, Tokyo, Japan) for 5 hours at room temperature. After decalcification, the heads were cryoprotected in 20% sucrose in PBS overnight at room temperature, embedded in OCT compound (Sakura Finetek, Torrance, CA, USA), and frozen in ethanol cooled with dry ice. Frozen blocks were stored at -80 °C until use.

Olfactory tissues were coronally sectioned at the level of the anterior border of the eyes, as previously described (35), into 8- or 14-μm-thick sections for Wright staining or other histological staining, respectively, using a cryostat (Leica Biosystems, Nussloch, Germany). Sections were mounted on glass slides, air-dried, and stored at -30 °C until staining.

### 2.3 Histological and immunohistochemical staining

For Wright staining, frozen sections were defatted in xylene and dehydrated through a graded ethanol series. Sections were then rehydrated in distilled water (DW), air-dried with cooled air, and stained with Wright staining solution (Muto Kagaku, Tokyo, Japan) for 10 seconds. The sections were rinsed with PBS for 20 seconds to stabilize the staining, washed with DW, air-dried with cooled air, cleared in xylene, and coverslipped with HSR mounting medium (Sysmex, Kobe, Japan).

For Alcian blue staining, frozen sections were defatted in xylene and dehydrated through a graded ethanol series. After rehydration in DW, the tissue sections were stained with Alcian blue solution (pH 2.5; Muto Kagaku) for 45 minutes, rinsed with 0.5% acetic acid followed by DW, counterstained with Kernechtrot for 1 minute, dehydrated through a graded ethanol series, cleared in xylene, and coverslipped with Marinol (Muto Kagaku).

For immunohistochemistry, frozen sections were rehydrated with TBST buffer [10 mM Tris-HCl (pH 7.4) and 100 mM NaCl with 0.1% Tween 20]. For antigen retrieval, sections were incubated in Tris- ethylenediaminetetraacetic acid (EDTA) buffer (pH 9.0) at 70 °C for 40 min and cooled to room temperature. The sections were blocked with blocking buffer (5% normal donkey serum in TBST) for 1 hour at room temperature and incubated overnight with primary antibodies diluted in blocking buffer. The primary antibodies used in the present study were rabbit anti-CD206 (ab300621, Abcam, Cambridge, UK), rabbit anti-Iba-1 (ab178846, Abcam), and rabbit anti-Glial Fibrillary acidic protein (GFAP) antibodies (IR524, Dako, Glostrup, Denmark). The sections were then incubated with a secondary antibody (ImmPress anti-rabbit IgG, Vector Laboratories, Burlingame, CA, USA) for 1 hour at room temperature and stained with ImmPACT DAB (Vector Laboratories). Nuclei were stained with Carrazzi’s hematoxylin for 10 sec. Finally, the slices were cleared and coverslipped with HSR.

Stained sections were observed using an Eclipse Ci-L microscope equipped with a digital camera control unit (DS-Fi3/NIS-Elements; Nikon, Tokyo, Japan).

### 2.4 Image analysis and morphometry

Images of CD206-immunostained sections were analyzed using Adobe Photoshop (Adobe Inc., San Jose, CA, USA). The total lamina propria area was manually delineated, and the CD206-positive regions within the delineated area were selected using the color selection tool in Adobe Photoshop. The CD206-positive area was expressed as a percentage of the total lamina propria area. Quantitative analyses were performed using two sections per mouse obtained from comparable anatomical levels in all animals (CONT, n = 5; ECRS, n = 5).

Similarly, Alcian blue-stained sections were analyzed using Adobe Photoshop. The total lamina propria area was manually delineated, and the Alcian blue-positive regions within the delineated area were selected using the color selection tool in Adobe Photoshop. The Alcian blue-positive area was expressed as a percentage of the total lamina propria area. Quantitative analyses were performed using two sections per mouse obtained from comparable anatomical levels in all animals (CONT, n = 5; ECRS, n = 4).

### 2.5 Tissue collection and bacterial DNA extraction

Mice were deeply anesthetized with ketamine and xylazine, and transcardially perfused with PBS. After decapitation, the skin, lower jaw, upper teeth, upper palate, brain, and skull were removed, and fresh olfactory tissues, including the nasal bone and nasal mucosa, were collected under a clean bench. The tissues were weighed, snap-frozen in liquid nitrogen, and stored at -80 °C until use for nasal microbiota analysis.

Bacterial DNA from nasal tissues was extracted using the QIAamp DNA Microbiome kit (QIAGEN, Hilden, Germany) according to the manufacturer’s protocol. After bacterial cells were isolated from nasal tissues (CONT, n = 7; ECRS, n = 9), the bacterial pellets were mechanically disrupted using FastPrep-24 5G (MP Biomedicals, Irvine, CA, USA) at 6.5 m/sec for 45 seconds, placed on ice for 5 min, then crushed again at 6.5 m/sec for 45 seconds. As a negative extraction control, sterile PBS was processed in parallel using the same DNA extraction and 16S rRNA gene sequencing procedures. The negative control yielded only a negligible number of sequence reads, indicating minimal background contamination during sample processing.

For gut microbiota analysis, mice were deeply anesthetized and perfused with PBS, after which the abdomen was opened and the cecal contents were freshly collected (CONT, n =9; ECRS, n = 11), weighed, snap-frozen in liquid nitrogen, and stored at -80 °C until use.

Bacterial DNA from the cecal contents was extracted using the QIAamp Power Fecal Pro DNA kit (QIAGEN) according to the manufacturer’s protocol. Cecal samples were homogenized using FastPrep-24 5G instrument (MP Biomedicals) at 6.0 m/sec for 40 sec.

After bacterial DNA extraction, the V3-V4 region of the 16S rRNA gene was amplified by PCR using universal primers (forward primer:

5’ -TCGTCGGCAGCGTCAGATGTGTATAAGAGACAGCCTACGGGNGGCWGCAG -3’; reverse primer: 5’ - GTCTCGTGGGCTCGGAGATGTGTATAAGAGACAGGACTACHVGGGTATCTAATCC -3’) and Platinum II Hot-Start PCR Master Mix (Invitrogen, Thermo Fisher Scientific, Waltham, MA, USA). PCR was performed under the following conditions: initial denaturation at 94 °C for 2 min, followed by 35 cycles for bacterial DNA from nasal tissues or 25 cycles for cecal DNA of denaturation at 94 °C for 30 s, annealing at 55 °C for 30 s, and extension at 68 °C for 45 s, with a final extension at 68 °C for 5 min. PCR products were purified using AMPure XP (Beckman Coulter, Brea, CA, USA) according to the manufacturer’s protocol. Indexed libraries were prepared using the Nextera XT Index Kit (Illumina, San Diego, CA, USA), and sequencing was performed on an Illumina NextSeq platform using paired-end reads.

### 2.6 16S rRNA gene sequencing and analysis

The 16S rRNA gene sequencing data were processed using the Quantitative Insights Into Microbial Ecology 2 (QIIME 2) pipeline (36). Raw paired-end sequence data were demultiplexed and quality-filtered using the q2-demux plugin, followed by denoising and merging of paired-end reads with amplicon sequence variant (ASV) inference using DADA2 plugin (37). Taxonomic classification of ASVs was performed using the q2-feature-classifier plugin with a naïve Bayes classifier (classify-sklearn) trained on the SILVA 138.1 reference database trimmed to the V3-V4 region. Taxonomic assignments were made with a confidence threshold of 0.7, and downstream analyses were conducted primarily at the genus level.

Within-sample diversity (α-diversity) was calculated using QIIME 2. Rarefaction curves were generated based on the Shannon diversity index. An even sequencing depth of approximately 22,000 reads per sample was used for the calculation of richness and diversity indices.

Between-sample diversity (β-diversity) was assessed by calculating Bray-Curtis distances using QIIME 2 default scripts. Principal coordinate analysis (PCoA) was applied to the resulting distance matrices to visualize differences in microbial community composition.

Differentially abundant taxa between the ECRS and CONT groups were identified using linear discriminant analysis effect size (LEfSe) (38). Taxa with a linear discriminant analysis (LDA) score > 2.0 and p < 0.05 were considered differentially abundant.

### 2.7 PICRUSt2 analysis

Microbial functional profiles were predicted from the 16S rRNA gene amplicon data using PICRUSt2 (version 2.5.3) (39). ASV were placed into a reference phylogeny using SATé-enabled phylogenetic placement (SEPP) (40), followed by prediction of gene-family abundances and reconstruction of MetaCyc pathway abundances. Differential pathway abundance between the ECRS and CONT groups was assessed, and p values were adjusted for multiple comparisons using the Benjamini-Hochberg false discovery rate (FDR) method to obtain q values. Pathways with q < 0.05 were considered significant, whereas pathways with 0.05 ≤ q < 0.10 were considered exploratory.

### 2.8 RNA extraction and quantitative RT-PCR

Mice were deeply anesthetized with ketamine and xylazine, and the OB and the hippocampus were rapidly dissected on ice, weighed, snap-frozen with liquid nitrogen, and stored at -80 °C until use. Total RNA was extracted from frozen brain tissues using a NucleoSpin RNA kit (Macherey-Nagel GmbH & C. KG, Düren, Germany) according to the manufacturer’s instructions. RNA concentration was determined by measuring absorbance at 260 nm. Complementary DNA (cDNA) was synthesized from 200 ng total RNA using the ReverTra Ace reverse transcription kit (TOYOBO, Osaka, Japan) following the manufacturer’s instructions.

Brain tissues from CONT (n = 13) and ECRS (n = 14) mice were used for quantitative real-time PCR analysis. Quantitative real-time PCR was performed using TaqMan Gene Expression Assays (Applied Biosystems-Thermo Fisher Scientific; **Table 1**) in a total reaction volume of 20 μL with the 7500 Fast Real-Time PCR System (Applied Biosystems-Thermo Fisher Scientific). The thermal cycling conditions were 40 cycles of denaturation at 95 °C for 3 s, followed by annealing/extension at 60 °C for 30 s. Gene expression levels were normalized to *Gapdh* as an internal control. Relative expression levels were calculated using the 2^-(ΔΔCT) method. Data are presented as fold changes relative to the control group. Error bars represent the standard deviation (SD) of the ΔΔCT values.

**Table 1:** TaqMan Probe.

Target	Assay ID
Gapdh	Mm99999915 g1
Aif1	Mm00479862 g1
Gfap	Mm01253033 m11
II-6	Mm00446190 m1
Tnf	Mm00443258 m1
II-4	Mm00445259 m1

### 2.9 MaAsLin2 analysis

Multivariable Associations with Linear Model 2 (MaAsLin2) was used to identify associations between microbial taxa and host gene expression levels. Relative abundance data of nasal and gut microbiota derived from 16S rRNA amplicon sequencing, together with gene expression values expressed as – ΔCt, were analyzed using MaAsLin2 implemented in R version 4.5.0 (41). Default parameters were used unless otherwise specified. Associations with a false discovery rate (FDR)-adjusted p value < 0.25 were considered statistically significant.

### 2.10 Statistics

Changes in body weight, food intake, and water intake were analyzed using two-way analysis of variance (ANOVA) with treatment (CONT vs. ECRS) and time (weeks 1, 2, and 3) as factors. Differences in the percentages of CD206-positive and Alcian blue-positive areas, alpha diversity indices, relative taxonomic abundances, and gene expression levels determined by quantitative real-time PCR were analyzed using the Mann-Whitney U test. Differences in microbial beta diversity were evaluated using permutational multivariate analysis of variance (PERMANOVA), and homogeneity of multivariate dispersion was assessed using permutational analysis of multivariate dispersions (PERMDISP). For these analyses, a p value < 0.05 was considered statistically significant.

## 3 Results

### 3.1 Characterization of the ECRS mouse model

During the 3-week treatment period to induce the ECRS model, we monitored several physiological parameters, including body weight, food intake, and water intake. Body weight gradually increased over the 3 weeks in both the ECRS and CONT groups, with no significant difference between groups (**Supplementary Figure 1A**). The 3-week average daily food intake was approximately 3.0 g in the ECRS group and 2.9 g in the CONT group, while water intake was 5.1 g and 4.7 g, respectively, and showed no significant differences between groups (**Supplementary Figures 1B, C**). These findings suggest that the ECRS model did not induce marked systemic physiological alterations.

**Figure 2:**
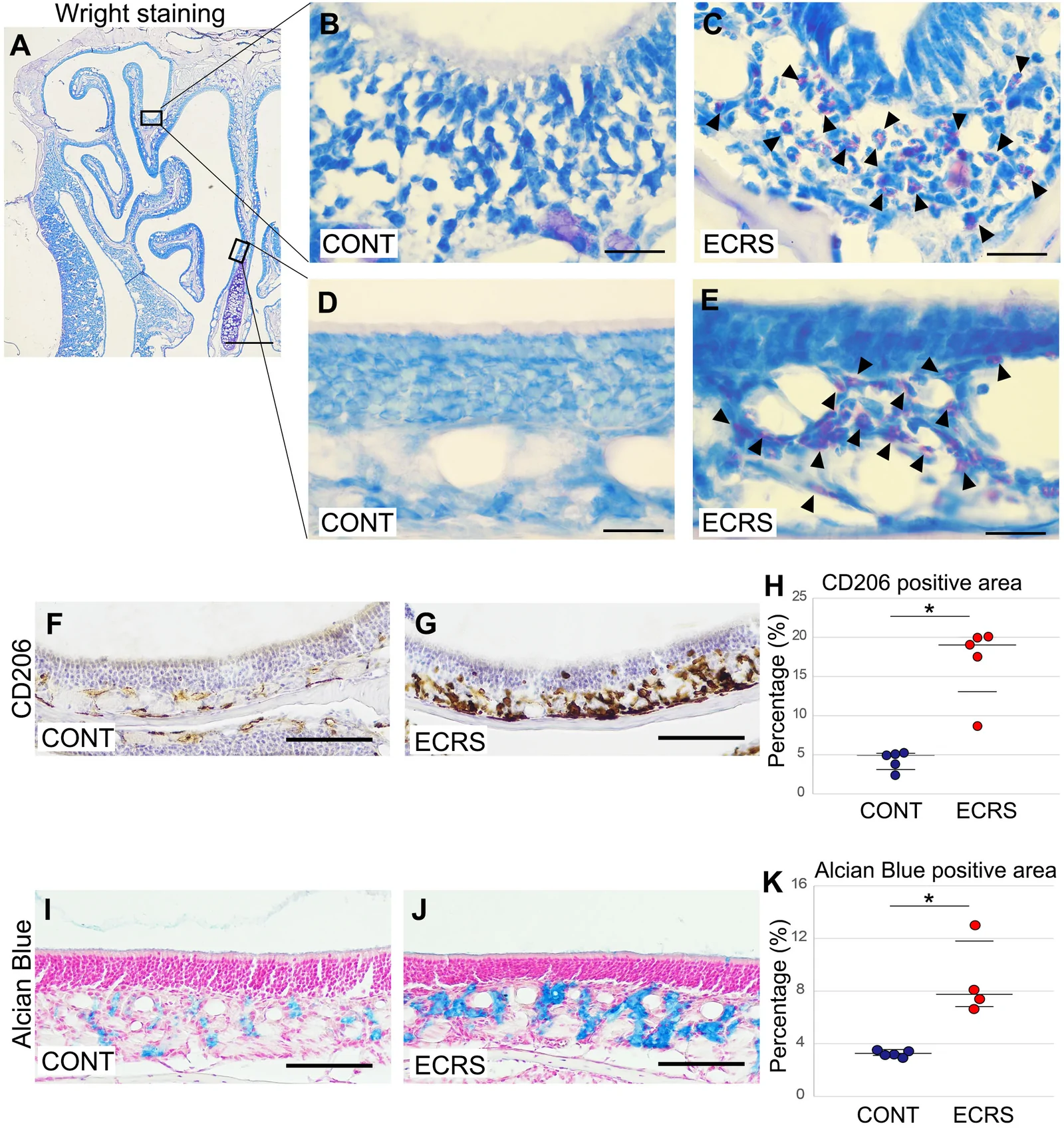
Eosinophilic infiltration and histopathological changes in the olfactory mucosa. **(A)** Wright staining of a coronal section of the olfactory mucosa from a representative control (CONT) mouse. The boxed areas indicate the regions shown at higher magnification in **(B–E)**. Scale bar, 500 μm. **(B–E)** Higher-magnification images of the corresponding regions in CONT **(B, D)** and ECRS **(C, E)** groups. Marked eosinophilic infiltration was observed in the ECRS group **(C, E)**, whereas such infiltration was rarely observed in the CONT group **(B, D)**. Arrowheads indicate representative eosinophils infiltrating the lamina propria. Scale bars, 20 μm. **(F, G)** Representative immunohistochemical images of CD206, an M2 macrophage marker, in the olfactory mucosa of the CONT **(F)** and ECRS **(G)** groups. CD206-positive areas were expanded in the lamina propria of the ECRS group. Scale bars, 100 μm. **(H)** Quantification of the CD206-positive area expressed as a percentage of the total lamina propria area (%). The CD206-positive area was significantly increased in the ECRS group (CONT, n = 5; ECRS, n = 5). **(I, J)** Representative Alcian blue staining of the olfactory mucosa in the CONT **(I)** and ECRS **(J)** groups. Alcian blue-positive areas were expanded in the lamina propria of the ECRS group. Scale bars, 100 μm. **(K)** Quantification of the Alcian blue-positive area expressed as a percentage of the total lamina propria area (%). The Alcian blue-positive area was significantly increased in the ECRS group (CONT, n = 5; ECRS, n = 4). Data were analyzed using the Mann-Whitney U test. *p < 0.05.

In contrast, Wright staining revealed marked eosinophilic infiltration in the olfactory mucosa of the ECRS group, but not in the CONT group (**Figures 2A–E**). Furthermore, immunohistochemistry demonstrated increased CD206-positive M2 macrophages (**Figures 2F–H**), and Alcian blue staining showed increased acidic mucin in the lamina propria of the ECRS group (**Figures 2I–K**). Together, these histopathological features confirm the successful establishment of the model.

### 3.2 Distinct microbial community structures in the nasal cavity and gut

**Figure 3:**
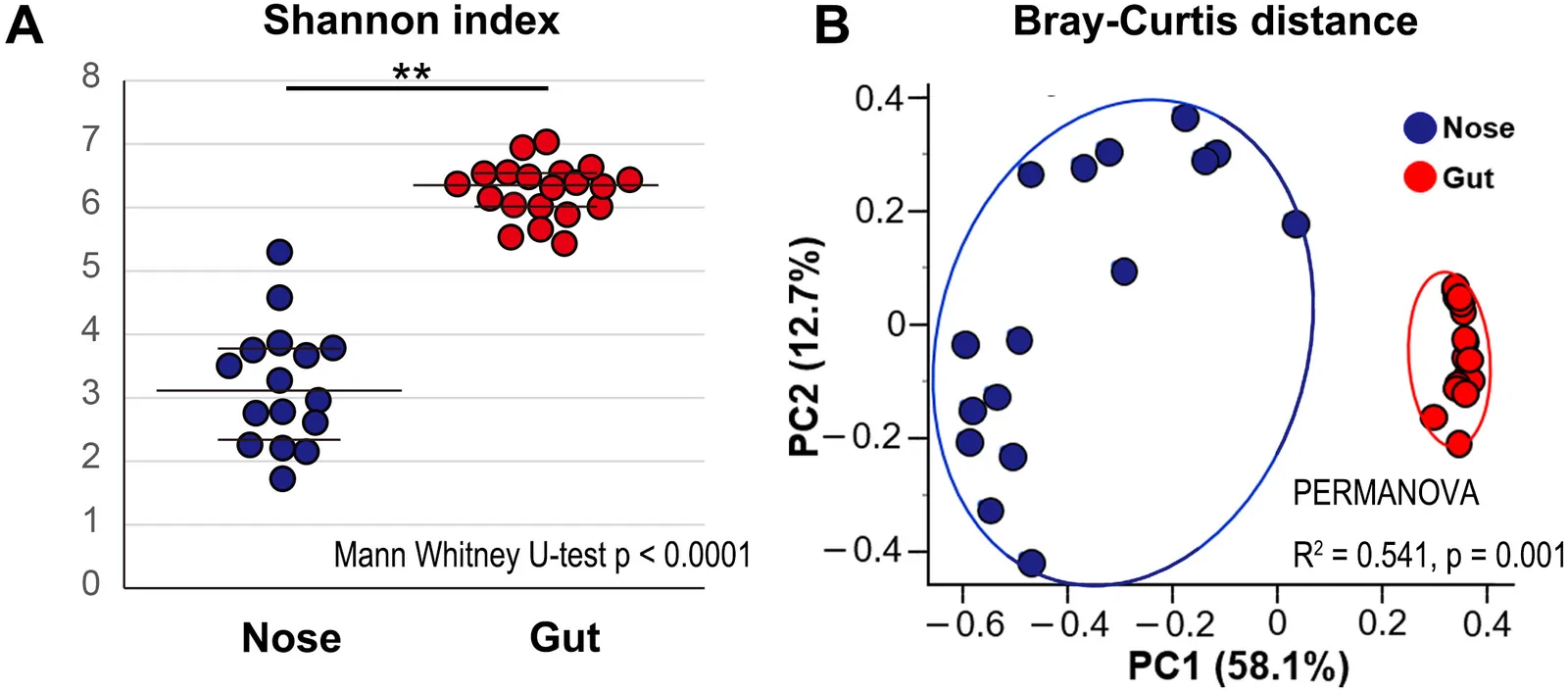
Differences in microbial community composition between the nasal and gut microbiota. **(A)** Alpha diversity of the nasal and gut microbiota assessed using the Shannon diversity index. Blue dots represent the nasal microbiota (n = 16; CONT, n = 7; ECRS, n = 9) and red dots represent the gut microbiota (n = 20; CONT, n = 9; ECRS, n = 11). Shannon diversity was significantly lower in the nasal microbiota than in the gut microbiota (Mann-Whitney U test) **p < 0.01. **(B)** Principal coordinate analysis (PCoA) based on Bray-Curtis distances showing beta diversity of the nasal and gut microbiota. PERMANOVA revealed a significant difference in microbial community composition between the nasal and gut microbiota (R^2^ = 0.541, p = 0.001).

To provide a baseline for interpreting ECRS-associated microbial changes, we first compared the overall microbial community structures of the nasal cavity and cecum. The Shannon diversity index was significantly lower in the nasal microbiota than in the gut microbiota (p < 0.0001, **Figure 3A**). Principal coordinate analysis based on Bray-Curtis dissimilarity demonstrated clear separation between the nasal and gut microbiota (PERMANOVA, R^2^ = 0.541, p = 0.001; **Figure 3B**). Consistent separation was also observed using weighted and unweighted UniFrac distance metrics (PERMANOVA, R^2^ = 0.620 and 0.658, respectively; both p = 0.001). Homogeneity of multivariate dispersion also differed significantly between the nasal and gut microbiota (PERMDISP, all p = 0.001), with greater within-group dispersion observed in the nasal microbiota than in the cecal microbiota. These findings indicate that the nasal and gut harbor distinct microbial communities.

### 3.3 Dysbiosis of nasal microbiota in ECRS

**Figure 4:**
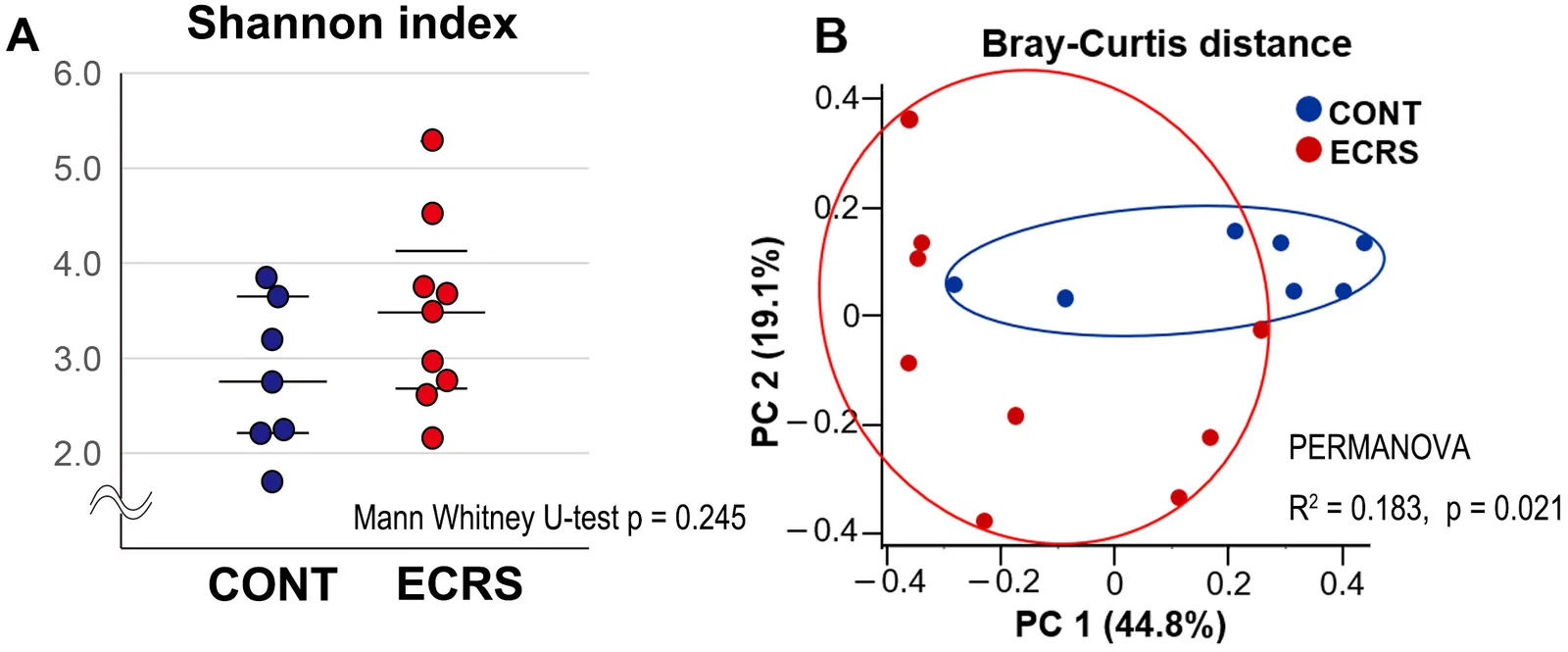
Alterations in nasal microbiota diversity in the ECRS group. **(A)** Alpha diversity of the nasal microbiota assessed using the Shannon diversity index in the control (CONT) (blue dots, n = 7) and ECRS (red dots, n = 9) groups. No significant difference was observed between groups (Mann-Whitney U test). **(B)** Principal coordinate analysis (PCoA) based on Bray-Curtis distances showing beta diversity of the nasal microbiota in the CONT (blue dots, n = 7) and ECRS (red dots, n = 9) groups. PERMANOVA revealed a significant difference in microbial community composition between groups (R^2^ = 0.183, p = 0.021).

Next, we analyzed differences in the diversity and composition of the nasal microbiota between the ECRS and CONT groups. The Shannon diversity index did not differ significantly between groups. In contrast, Bray-Curtis distance analysis revealed a significant difference in overall nasal microbial community composition between the ECRS and CONT groups (PERMANOVA, R^2^ = 0.183, p = 0.021). No significant difference in multivariate dispersion was detected between groups (PERMDISP, p = 0.154), indicating that the observed community difference was not attributable to differences in within-group dispersion (**Figure 4**).

**Figure 5:**
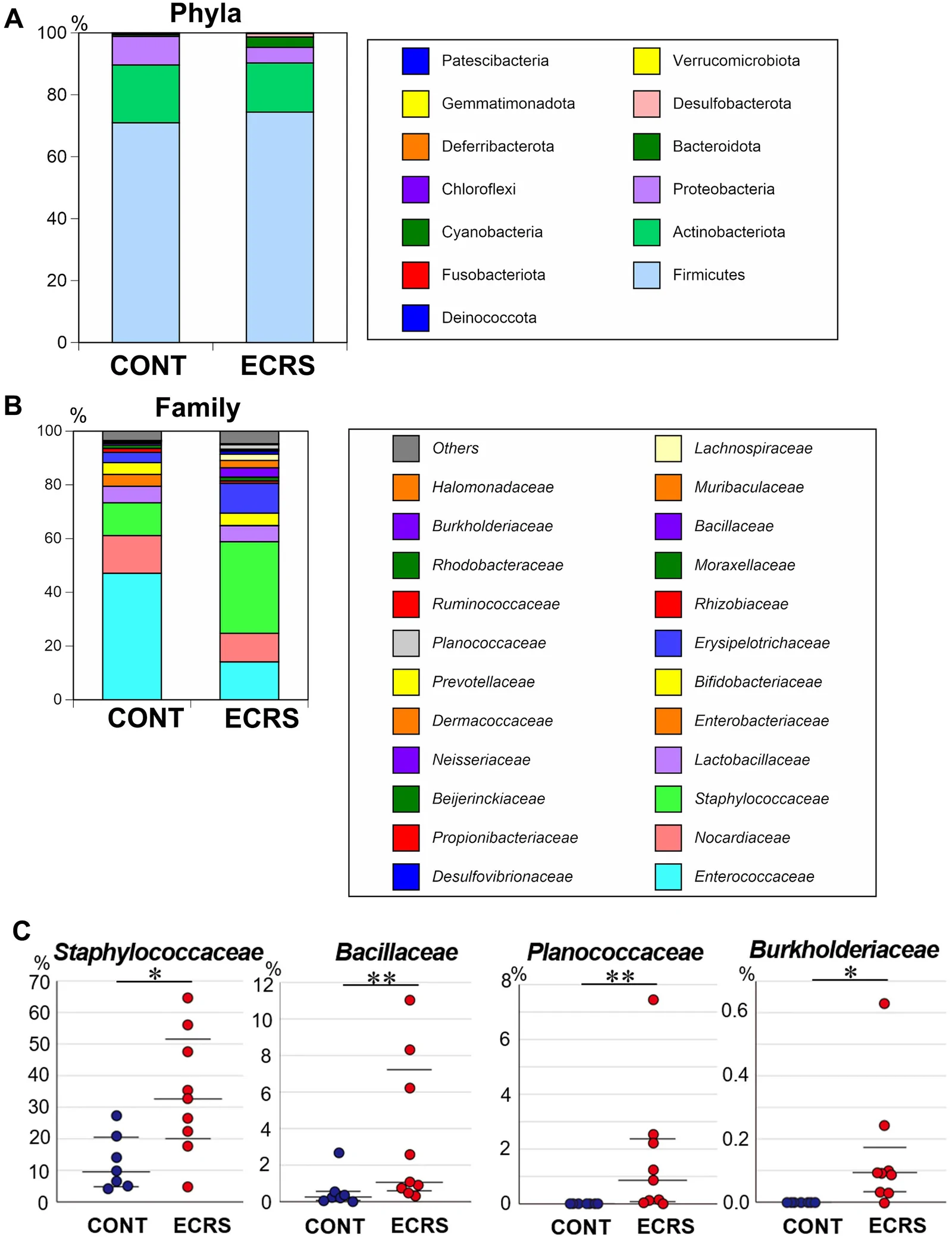
Alterations in nasal microbiota composition in the ECRS group. **(A)** Microbial community composition at the phylum level. The relative abundances of the major phyla did not differ between the ECRS and control (CONT) groups. More than 90% of the nasal microbiota consisted of Firmicutes, Actinobacteriota, and Proteobacteria in both groups. **(B)** Microbial community composition at the family level. **(C)** Families showing significantly altered relative abundance in the ECRS group compared with the CONT group. The relative abundances of *Staphylococcaceae*, *Bacillaceae*, *Planococcaceae*, and *Burkholderiaceae* were increased in the ECRS group (Mann-Whitney U test). *p < 0.05, **p < 0.01. CONT, n = 7; ECRS, n = 9.

At the phylum level, no significant differences in relative abundance were observed between the ECRS and CONT groups (**Figure 5A**). At the family level, *Staphylococcaceae*, *Bacillaceae*, *Planococcaceae*, and *Burkholderiaceae* were significantly enriched in the ECRS group. *Ruminococcaceae*, *Halomonadaceae*, *Beijerinckiaceae*, *Dermacoccaceae*, *Rhodobacteraceae*, and *Propionibacteriaceae* also showed a tendency toward higher abundance, whereas *Enterococcaceae* tended to be less abundant in the ECRS group (**Figures 5B, C**; **Supplementary Table 1**). At the genus level, *Staphylococcus, Bacillus, Sporosarcina*, and *Burkholderia_Caballeronia_Paraburkholderia* were significantly enriched in the ECRS group (**Supplementary Figure 2**).

**Figure 6:**
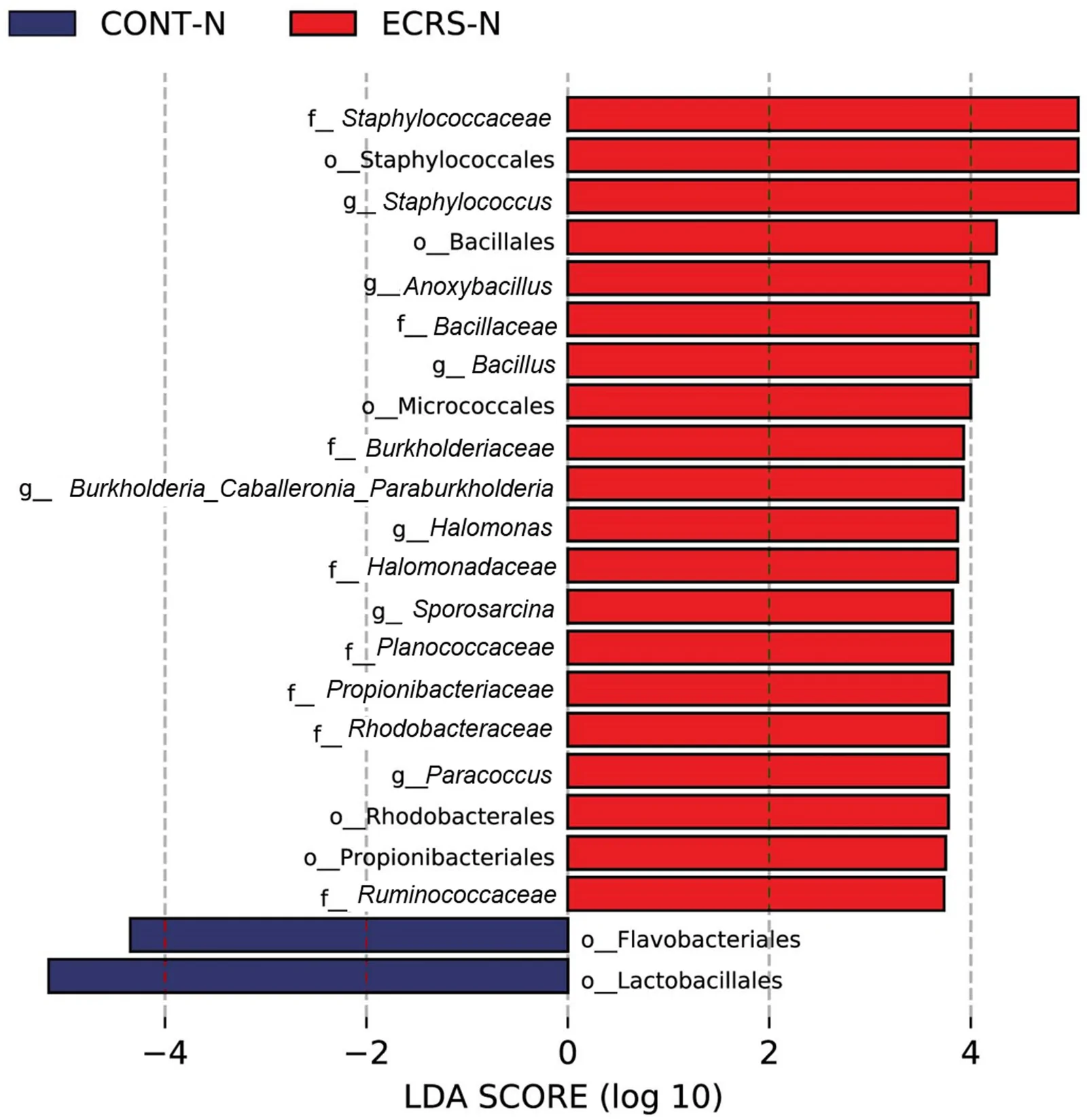
Differentially abundant nasal bacterial taxa between the ECRS and CONT groups. The suffix “N” denotes nasal microbiota. Nasal bacterial taxa showing significantly different relative abundances between the ECRS-N (red) and CONT-N (blue) groups, as identified by LEfSe. Taxa with a linear discriminant analysis (LDA) score > 2 are shown.

Consistent with these findings, LEfSe analysis identified bacterial taxa that differed significantly in relative abundance based on LDA scores. The families *Staphylococcaceae*, *Bacillaceae*, *Planococcaceae*, *Burkholderiaceae*, *Ruminococcaceae*, *Halomonadaceae*, *Rhodobacteraceae*, and *Propionibacteriaceae* were enriched in the ECRS group, whereas the orders Flavobacteriales and Lactobacillales were enriched in the CONT group (**Figure 6**).

### 3.4 Dysbiosis of gut microbiota in ECRS

**Figure 7:**
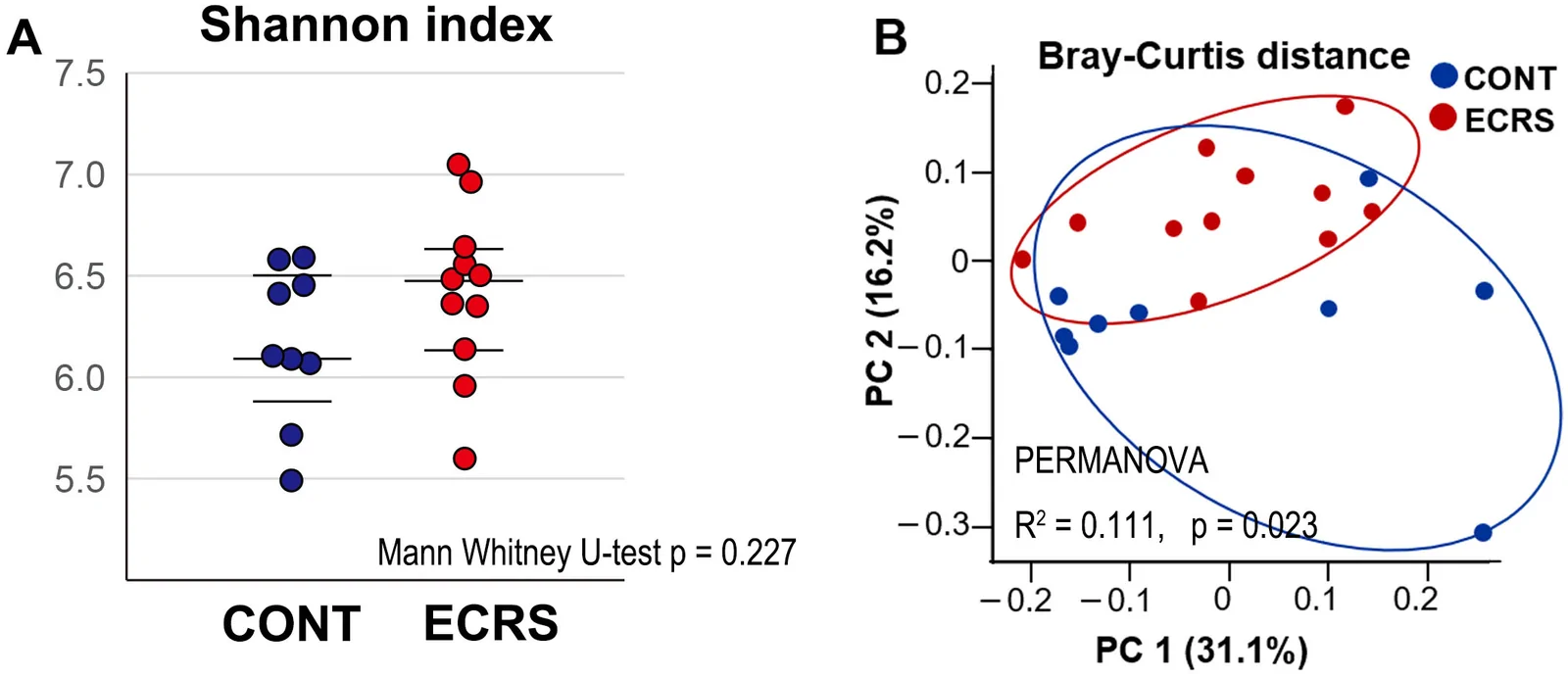
Alterations in gut microbiota diversity in the ECRS group. **(A)** Alpha diversity of the gut microbiota assessed using the Shannon diversity index in the control (CONT) (blue dots, n = 9) and ECRS (red dots, n = 11) groups. No significant difference was observed between groups (Mann-Whitney U test). **(B)** Principal coordinate analysis (PCoA) plot based on Bray-Curtis distances showing beta diversity of the gut microbiota in the CONT (blue dots, n = 9) and ECRS (red dots, n = 11) groups. PERMANOVA revealed a significant difference in microbial community composition between groups (R^2^ = 0.111, p = 0.023).

We then analyzed differences in the diversity and composition of the gut microbiota between the ECRS and CONT groups. The Shannon diversity index did not differ significantly between groups. In contrast, Bray-Curtis distance analysis revealed a significant difference in overall microbial community composition between the ECRS and CONT groups (PERMANOVA, R^2^ = 0.111, p = 0.023). No significant difference in multivariate dispersion was detected between the groups (PERMDISP, p = 0.247), indicating that the observed difference in community composition was not attributable to differences in within-group dispersion (**Figure 7**). However, no significant differences between the ECRS and CONT groups were detected using weighted or unweighted UniFrac distances metrics.

**Figure 8:**
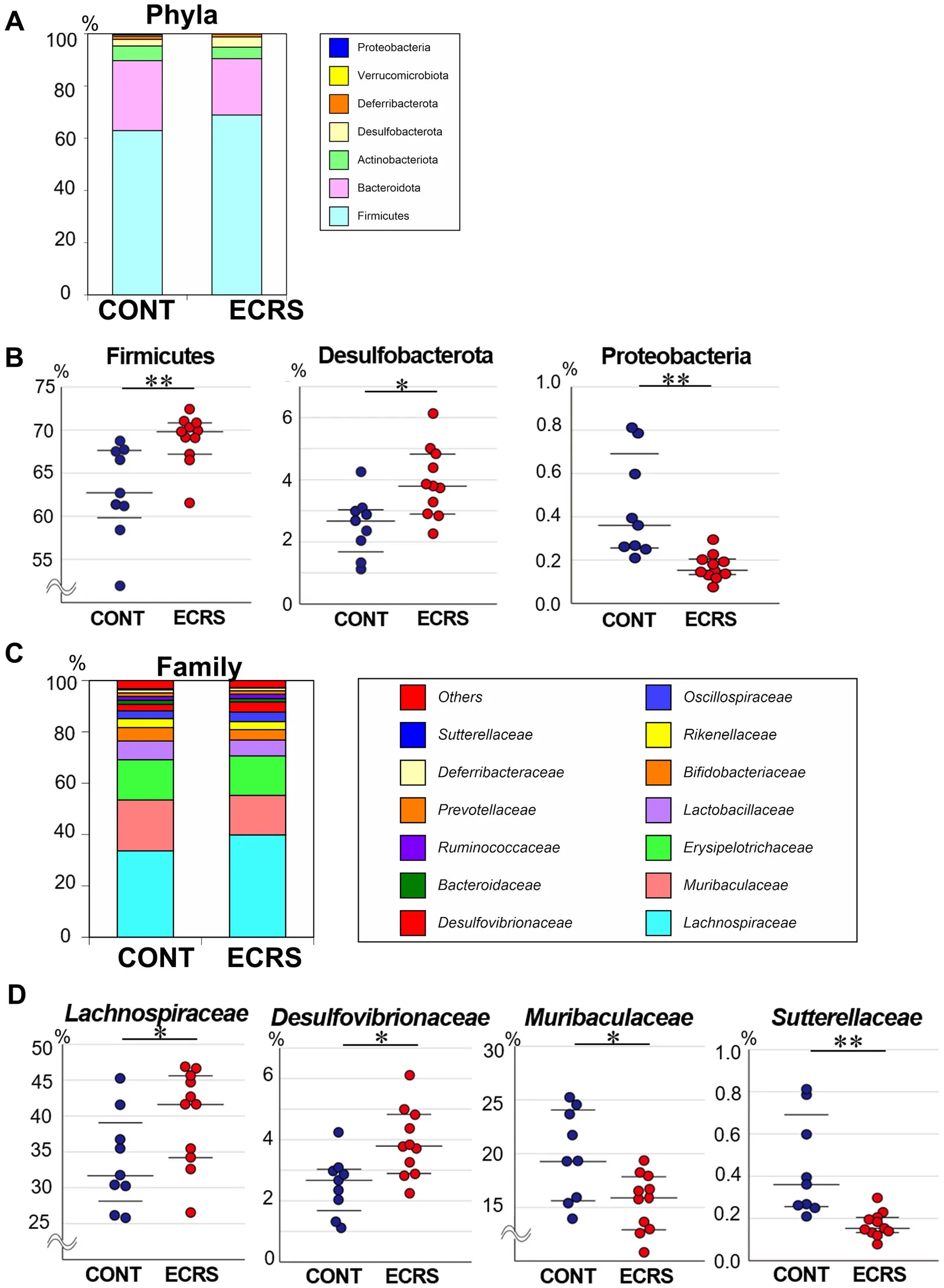
Alterations in gut microbiota composition in the ECRS group. **(A)** Microbial community composition at the phylum level. **(B)** Phyla showing significantly altered relative abundance in the ECRS group (red dots, n = 11) compared with the control (CONT) group (blue dots, n = 9). The relative abundances of Firmicutes and Desulfobacterota were increased, whereas that of Proteobacteria was decreased in the ECRS group (Mann-Whitney U test). *p < 0.05, **p < 0.01. **(C)** Microbial community composition at the family level. **(D)** Families showing significantly altered relative abundance in the ECRS group (red dots, n = 11) compared with the CONT group (blue dots, n = 9). The relative abundances of *Lachnospiraceae* and *Desulfovibrionaceae* were increased, whereas those of *Muribaculaceae* and *Sutterellaceae* were decreased in the ECRS group (Mann-Whitney U test). *p < 0.05, **p < 0.01.

At the phylum level, Firmicutes and Desulfobacterota were significantly enriched, whereas Proteobacteria was significantly depleted in the ECRS group (**Figures 8A, B**). At the family level, *Lachnospiraceae* and *Desulfovibrionaceae* were significantly more abundant, whereas *Muribaculaceae* and *Sutterellaceae* were significantly less abundant in the ECRS group (**Figures 8C, D**). At the genus level, *Roseburia, Tyzzerella*, *Desulfovibrio*, and *Ruminococcaceae*_UBA1819 were significantly more abundant, whereas *Parasutterella*, and the genus level taxa *Muribaculaceae* and *Rikenellaeae*_RC9_gut_group were significantly less abundant in the ECRS group (**Supplementary Figure 3**).

**Figure 9:**
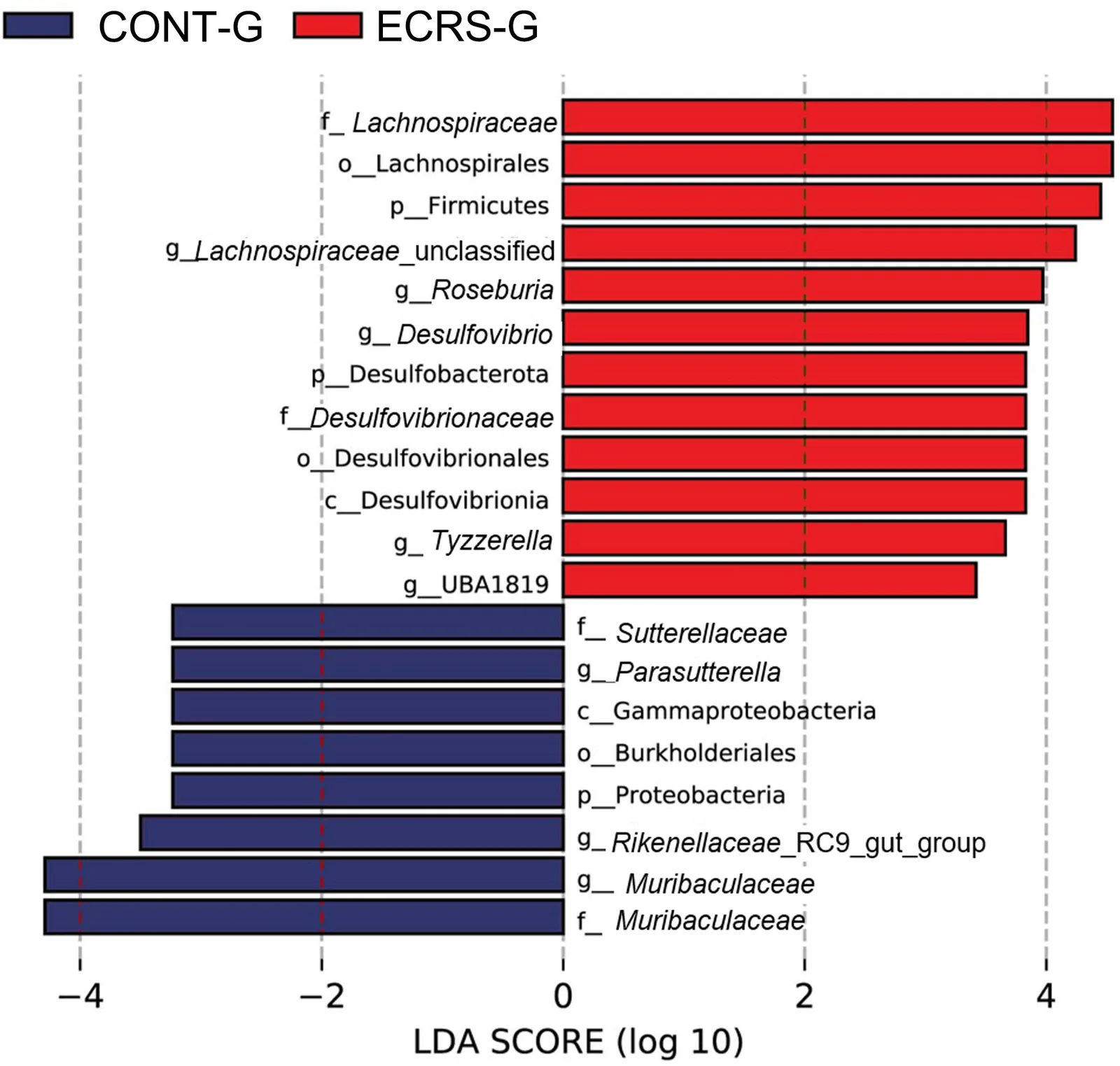
Differentially abundant gut microbial taxa between the ECRS and CONT groups. The suffix “G” denotes gut microbiota. Gut bacterial taxa showing significantly different relative abundances between the ECRS (red) and CONT (blue) groups, as identified by LEfSe. Taxa with a linear discriminant analysis (LDA) score > 2 are shown.

Consistent with these findings, LEfSe analysis identified the phyla Firmicutes and Desulfobacterota, as well as the families *Lachnospiraceae* and *Desulfovibrionaceae*, as enriched in the ECRS group, whereas the phylum Proteobacteria and the families *Muribaculaceae* and *Sutterellaceae* were enriched in the CONT group (**Figure 9**).

### 3.5 Predicted microbial functions

We performed PICRUSt2 analysis to infer microbial functional potential from the 16S rRNA gene profiles (**Supplementary Table 2**). In the nasal microbiota, seven predicted MetaCyc pathways differed between the ECRS and CONT groups at q < 0.10, of which four met the more stringent threshold of q < 0.05: the urea cycle, the superpathway of sulfur oxidation, formaldehyde assimilation II, and formaldehyde oxidation I. All seven pathways showed higher predicted abundances in the ECRS group. In addition, the predicted MetaCyc pathway “Peptidoglycan biosynthesis II (staphylococci)” was identified at the exploratory threshold of q < 0.10. In contrast, no predicted MetaCyc pathways differed significantly between the ECRS and CONT groups in the gut microbiota after multiple testing correction.

### 3.6 Glial activation and inflammatory gene expression in the brain

To investigate neuroinflammatory changes, we evaluated glial activation by immunohistochemistry for Iba-1 and GFAP, and by quantitative PCR for *Aif-1*, *Gfap*, *Il-6*, *Tnf*, and *Il-4* in the OB and hippocampus.

**Figure 10:**
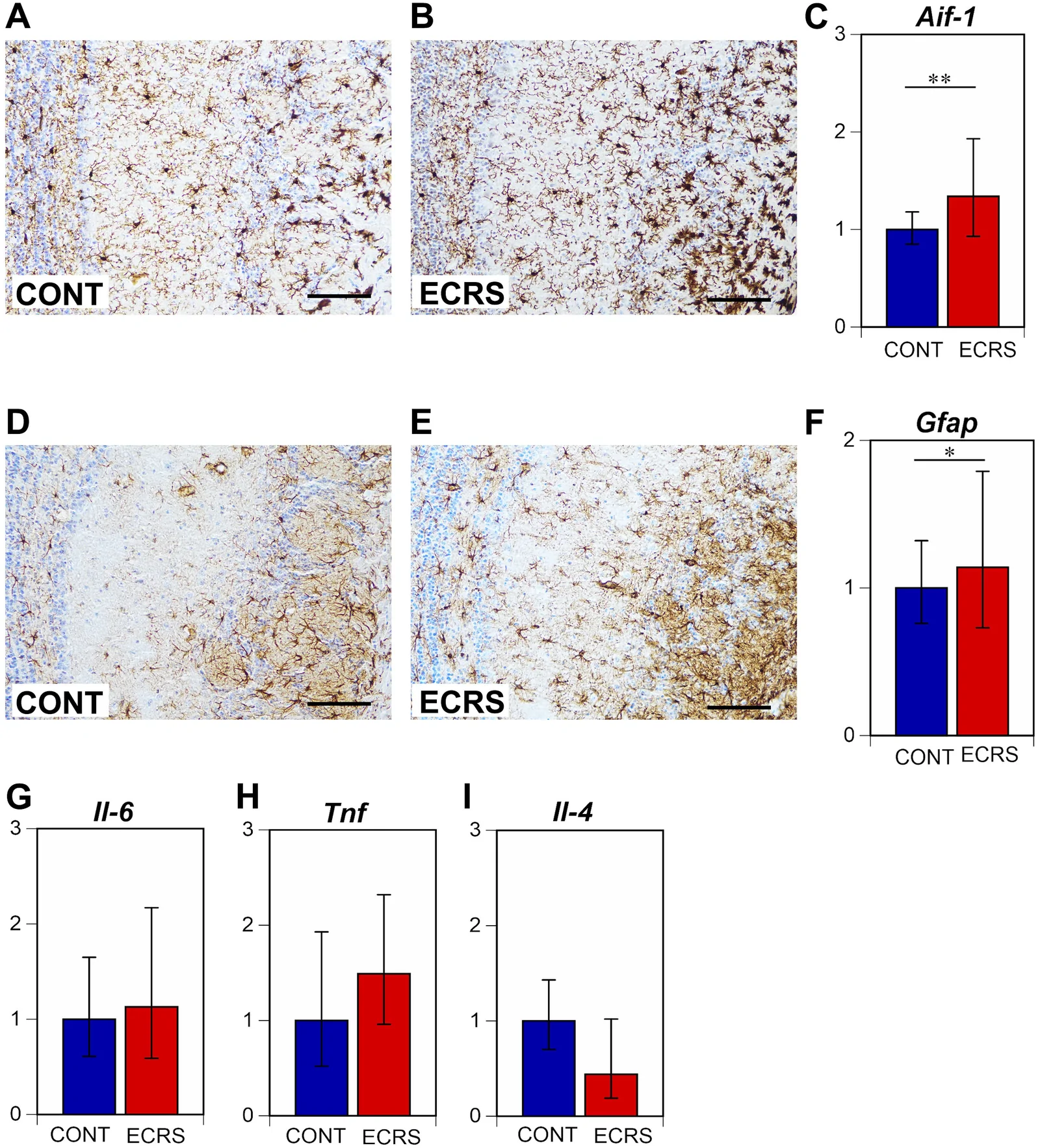
Neuroimmune alterations in the olfactory bulb. **(A, B)** Representative immunohistochemical images of Iba-1 in the olfactory bulb (OB). Microglia showed increased activation in the ECRS group **(B)** compared with the control (CONT) group **(A)**. Scale bars, 100 μm. **(C)** Quantitative PCR showed that *Aif-1* expression was significantly increased in the ECRS group (red) compared with the CONT (blue) group (Mann-Whitney U test). **(D, E)** Representative immunohistochemical images of GFAP in the OB. GFAP immunoreactivity was distributed over a wider area in the ECRS group **(E)** than in the CONT group **(D)**. Scale bars, 100 μm. **(F)** Quantitative PCR showed that *Gfap* expression was significantly increased in the ECRS group compared with the CONT group (Mann-Whitney U test). **(G–I)** Quantitative PCR showed that the expression levels of *Il-6* **(G)**, *Tnf* **(H)**, and *Il-4* **(I)** did not differ between the ECRS and CONT groups. **p < 0.01; *p < 0.05. CONT, n = 13; ECRS, n = 14.

In the OB, immunohistochemical staining for Iba-1 showed activated microglial morphology in the ECRS group (**Figures 10A, B**). Consistent with these findings, quantitative PCR demonstrated a slight but significant increase in *Aif-1* expression in the ECRS group (**Figure 10C**). Similarly, GFAP Immunostaining showed increased astrocytic immunoreactivity in the OB, accompanied by significantly higher *Gfap* expression (**Figures 10D–F**). In contrast, the expression of the cytokines, *Il-6*, *Tnf*, and *Il-4*, did not differ significantly between groups (**Figures 10G–I**).

**Figure 11:**
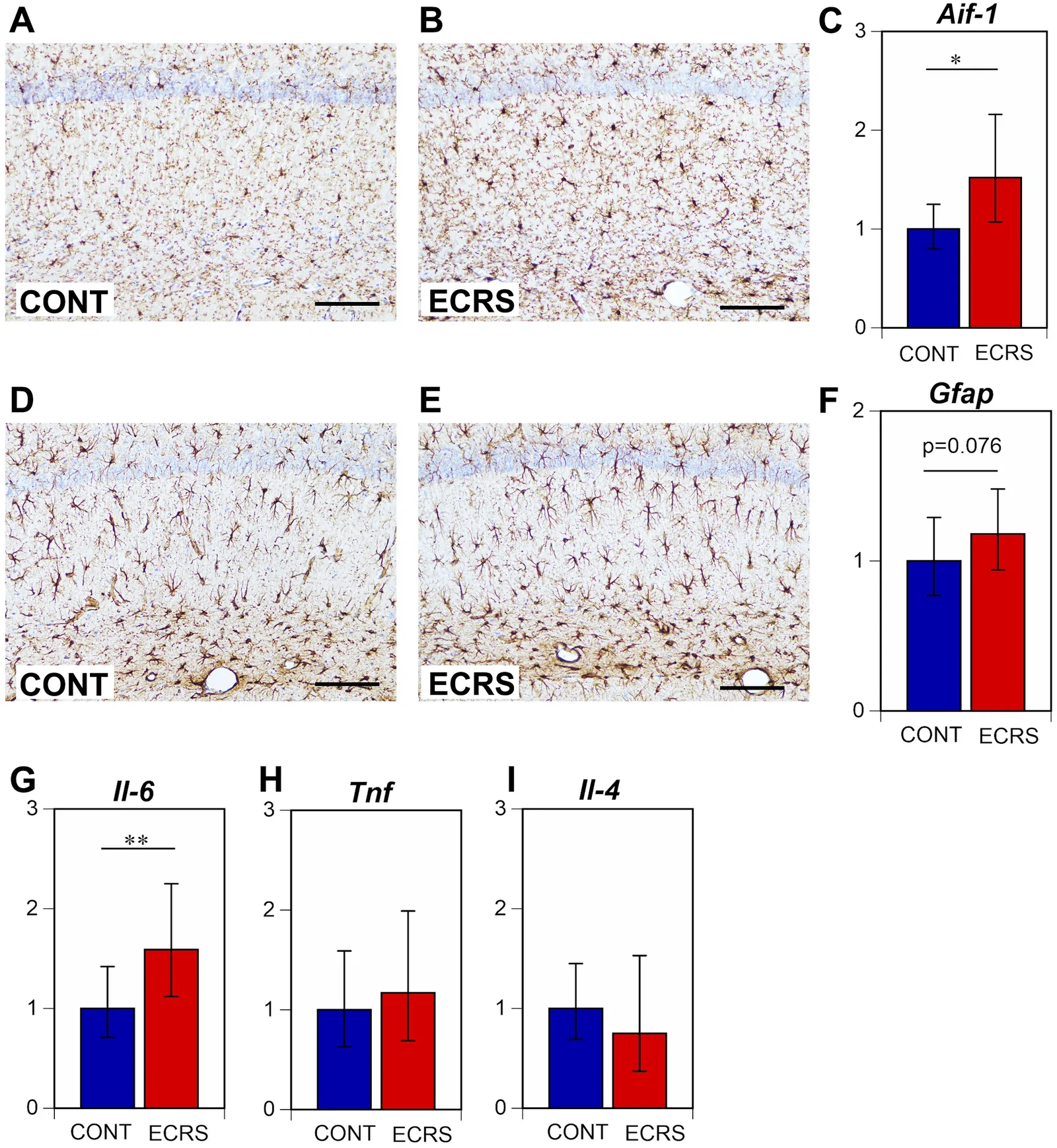
Neuroimmune alterations in the hippocampus. **(A, B)** Representative immunohistochemical images of Iba-1 in the hippocampus. Microglia appeared mildly activated in the ECRS group **(B)** compared with the control (CONT) group **(A)**. Scale bars, 100 μm. **(C)** Quantitative PCR showed that *Aif-1* expression was significantly increased in the ECRS group compared with the CONT group (Mann-Whitney U test). **(D, E)** Representative immunohistochemical images of GFAP in the hippocampus. GFAP immunoreactivity appeared similar between the ECRS **(E)** and CONT **(D)** groups. Scale bars, 100 μm. **(F)** Quantitative PCR showed that *Gfap* expression tended to be increased in the ECRS group compared with the CONT group (Mann-Whitney U test). **(G-I)** Quantitative PCR showed that *Il-6* expression **(G)** was significantly increased in the ECRS group, whereas the expression levels of *Tnf* **(H)** and *Il-4* **(I)** did not differ between the ECRS and CONT groups **p < 0.01; *p < 0.05.. CONT, n = 13; ECRS, n = 14.

In the hippocampus, microglia appeared mildly activated, and *Aif-1* expression was significantly increased in the ECRS group (**Figures 11A–C**). In contrast, no obvious difference in GFAP immunoreactivity was observed, although *Gfap* expression tended to be higher in the ECRS group (**Figures 11D–F**). In addition, *Il-6* expression was significantly increased in the ECRS group, whereas *Tnf* and *Il-4* expression did not differ significantly between groups (**Figures 11G–I**).

### 3.7 Association between microbiota and brain gene expression

Finally, we examined the associations between the microbiota and brain gene expression using MaAsLin2. In the OB, only one gut bacterial family showed a significant association with gene expression: the relative abundance of *Christensenellaceae* was positively associated with *Tnf* expression (**Supplementary Figure 4A**). No nasal bacterial taxa were significantly associated with gene expression in the OB.

In contrast, several nasal bacterial families were significantly associated with gene expression in the hippocampus. The relative abundances of *Rhodobacteraceae*, *Dermacoccaceae*, and *Neisseriaceae* were positively associated with *Aif-1* expression (**Supplementary Figures 4B–D, S5**). The relative abundances of *Neisseriaceae* and *Pseudomonadaceae* were positively associated with *Gfap* expression, whereas *Monoglobaceae* showed a negative association (**Supplementary Figures 4E-G, S5**). In addition, the relative abundances of *Rhodobacteraceae*, *Dermacoccaceae*, *Pseudomonadaceae*, *Thermaceae*, *Staphylococcaceae*, and *Xanthomonadaceae* were positively associated with *Il-6* expression in the hippocampus (**Supplementary Figure 4H-M, S5**). No gut bacterial taxa were significantly associated with gene expression in the hippocampus.

The bacterial taxa identified in this study, together with their microbiological characteristics, alteration in ECRS, and association with brain gene expression identified by MaAsLin2, are summarized in **Supplementary Table 3**.

## 4 Discussion

### 4.1 Summary of this study

In the present study, we first demonstrated that the nasal microbiota is compositionally distinct from the gut microbiota. We then showed that ECRS induces dysbiosis in both the nasal and gut microbiota, accompanied by altered expression of glial markers and inflammatory cytokine genes in the OB and hippocampus. MaAsLin2 analysis further revealed significant associations between nasal bacterial taxa and hippocampal glial and inflammatory gene expression, whereas associations between gut microbiota and hippocampal gene expression, as well as between nasal microbiota and OB gene expression, were limited. These findings suggest that ECRS-induced alterations in the nasal microbiota may be linked to hippocampal neuroimmune alterations.

### 4.2 Dysbiosis of nasal microbiota in ECRS

We first demonstrated that the nasal microbiota was compositionally distinct from the gut microbiota, forming clearly separated communities across all beta-diversity metrics. The differences between the nasal and gut microbiota were substantially greater than the microbial changes induced by ECRS. ECRS induced more subtle alterations within the nasal microbial community, which were detected by Bray-Curtis dissimilarity but not by weighted or unweighted UniFrac distances. These findings suggest that ECRS primarily alters the relative abundance of existing nasal bacterial taxa within an already distinct microbial ecosystem, rather than fundamentally reshaping the overall community structure. The greater within-group dispersion of the nasal microbiota compared with the cecal microbiota suggests that the nasal microbial community exhibits greater inter-individual variability, which may reflect its direct exposure to the external environment.

Recently, an increasing number of studies have investigated the nasal microbiota in patients with ECRS (22–24). However, there is no consistent conclusion regarding alpha diversity. Some studies reported increased alpha diversity as assessed by Chao1 and Shannon diversity indices (24), and others demonstrated decreased diversity based on observed OTUs, Chao1 indices (22), or Faith’s phylogenetic diversity (23). In addition, bacterial taxa showing altered abundance in ECRS patients vary among reports. These discrepancies may be attributed to unavoidable factors such as differences in sex, age, ethnicity, lifestyle, and analytical methods. In contrast, alterations in beta diversity have been consistently reported in ECRS patients, suggesting that ECRS consistently alters the overall composition of the nasal microbiota regardless of baseline microbial diversity. Consistent with these human studies, our results demonstrated a significant alteration in beta diversity in the ECRS group. Notably, the relative abundance of the family *Staphylococcaceae* was significantly increased, whereas that of the order Lactobacillales was decreased.

Members of the family *Staphylococcaceae* are common inhabitants of the human nasal cavity and include both commensal and opportunistic pathogenic species. They are facultatively anaerobic, catalase-positive, Gram-positive cocci. Therefore, they may have a selective advantage in the nasal cavities under conditions of reduced oxygen tension, potentially caused by nasal congestion. In addition, catalase activity may further enhance their survival under inflammatory conditions by neutralizing hydrogen peroxide generated by infiltrating immune cells, thereby conferring resistance to oxidative stress. *Staphylococcus aureus*, a representative species within this family, has been implicated in the pathogenesis of ECRS through the production of enterotoxins (42). Importantly, *S. aureus* preferentially colonizes and persists in tissues with impaired epithelial barrier function, such as inflamed skin (43). In such an inflammatory environment, *S. aureus* can evade host immune responses, including neutrophil-mediated clearance (44), and form biofilms (45), thereby promoting chronic inflammation. These characteristics of *S. aureus*, together with the general physiological properties of members of the family *Staphylococcaceae*, suggest that some members of this family may be well adapted to inflammatory and barrier-disrupted environments rather than being primary inducers of barrier damage. Although *S. aureus* itself was not detected in the present study due to the use of SPF mice, the expansion of *Staphylococcaceae* may reflect their adaptation to the eosinophilic inflammatory milieu induced by ECRS.

In contrast, members of the order Lactobacillales are common inhabitants of mucosal surfaces and include several bacterial groups with reported barrier-protective properties. In particular, strains belonging to *Lactobacilli* have been shown to protect epithelial tight junction structures and attenuate pathogen-induced disruption *in vitro* (46). Some *Lactobacilli* also enhance the expression and distribution of tight junction proteins such as ZO-1 and occludin (47), and prevent cytokine-induced increases in epithelial permeability (48). Although these effects cannot be generalized to all members of Lactobacillales, a reduction in Lactobacillales abundance may reflect a loss of bacterial taxa with potentially beneficial effects on mucosal barrier function.

### 4.3 Predicted microbial function

PICRUSt2 analysis provided additional evidence that the taxonomic alterations in the nasal microbiota were accompanied by changes in predicted microbial functional potential. Significant differences in predicted MetaCyc pathways were identified in the nasal microbiota between the ECRS and CONT groups, whereas no significant pathway-level differences were detected in the cecal microbiota despite the taxonomic shifts observed by LEfSe. This contrast may reflect functional redundancy among intestinal bacteria, greater interindividual variability in the cecal microbiota, or a greater sensitivity to functional shift in the nasal microbiota.

Among the nasal findings, the urea cycle and pathways related to sulfur oxidation and formaldehyde metabolism were enriched in the ECRS group and may reflect altered microbial metabolism associated with the inflammatory nasal environment. In addition, a staphylococcal-type peptidoglycan biosynthesis pathway showed an exploratory increase (0.05 ≤ q < 0.10), consistent with the increased abundance of *Staphylococcaceae*. Because PICRUSt2 predicts functional potential rather than actual metabolic activity, these observations should be regarded as hypothesis-generating and require validation by shotgun metagenomics or metabolomic analyses.

Taken together, the taxonomic, predicted functional, and brain gene expression analyses collectively suggest that alterations in the nasal microbiota were more closely associated with brain neuroimmune changes than alterations in the cecal microbiota. Nevertheless, the cross-sectional associations observed in this study do not establish causality, and future experimental studies will be needed to identify the microbial functions and mediators involved.

### 4.4 Gene expression changes in the OB and hippocampus

In the present study, the expression of glial marker genes was increased in both the OB and hippocampus, whereas *Il-6* was upregulated only in the hippocampus. These findings suggest a shift toward a low-grade inflammatory state in both brain regions in the ECRS group. Consistent with our findings, a recent study using the same murine ECRS model demonstrated increased microglial/macrophage and astrocyte accumulation in the OB by immunohistochemical analysis (49).

Accumulating evidence indicates that nasal inflammatory diseases can influence hippocampal neuroimmune status. Allergic rhinitis has been shown to induce neuroinflammation in the hippocampus and to elicit anxiety- and depression-like behaviors in rodents (9). Another study indicated that allergic rhinitis in rats was associated with upregulation of the microglial marker CD11b, the astrocytic marker GFAP, and inflammatory signaling in the hippocampus (50). In a mouse model of CRS, hippocampal neuronal damage and microglial activation mediated by HMGB1-dependent Notch1/Hes-1 signaling were observed, accompanied by neuroinflammation and anxiety- and depression-like behaviors (12). Consistent with these observations, our previous study using a hamster model of SARS-CoV-2 infection demonstrated that damage to the olfactory mucosa was accompanied by microglial and astrocytic activation in the hippocampus, together with a reduction in spine density of apical dendrites of hippocampal neurons at 42 days post-infection, a time point when acute inflammation had largely subsided (51). Collectively, these findings support the concept that nasal inflammation can induce neuroimmune alterations in the hippocampus.

In this context, the increased expression of glial marker genes together with elevated *Il-6* expression in the hippocampus may reflect neuroimmune alterations associated with eosinophilic nasal inflammation in this experimental model and may have relevance to brain functions involved in emotional and cognition. Although behavioral outcomes were not assessed in the present study, the observed hippocampal neuroimmune alterations suggest a potential impact of ECRS on brain function, including emotional regulation and cognition.

### 4.5 Association between nasal microbiota and hippocampal gene expression

In the present study, we identified differences in nasal and gut microbiota, as well as glial and inflammatory gene expression in the OB and hippocampus, between the ECRS and CONT groups. We then examined associations between microbiota composition and brain gene expression using MaAsLin2 (**Supplementary Figures 4, S5**).

Interestingly, statistically significant associations were detected between nasal microbiota and hippocampal gene expression, whereas no such associations were observed between nasal microbiota and the OB, or between gut microbiota and the hippocampus. This pattern suggests that alterations in nasal microbiota may be more closely associated with hippocampal neuroimmune changes than alterations in gut microbiota or gene expression in the OB. In the present model, inflammation was initiated locally in the nasal cavity; therefore, alterations in nasal microbiota may have been more prominent and more directly linked to disease-associated neuroimmune changes. Conversely, the absence of significant associations between gut microbiota and hippocampal gene expression suggests that, in the present model, gut microbiota alterations may be less directly associated with hippocampal neuroimmune changes. Because inflammation was experimentally induced in the nasal cavity, the present model does not allow determination of whether alterations in the nasal microbiota, gut microbiota, and hippocampal neuroimmune status occur sequentially or through bidirectional interactions. Addressing this question will require longitudinal and mechanistic studies.

Microbiota-host association analyses have been widely used to identify candidate microbial signatures that reflect host physiological or pathological status (52–55). In the present study, the nasal bacterial families *Staphylococcaceae*, *Dermacoccaceae*, and *Rhodobacteraceae* were significantly increased or tended to be increased in the ECRS group based on the Mann-Whitney U test and/or LEfSe analyses and were positively associated with hippocampal glial markers and an inflammatory cytokine by MaAsLin2 analysis. In contrast, the families *Neisseriaceae*, *Monoglobaceae*, *Pseudomonadaceae*, *Thermaceae*, and *Xanthomonadaceae* did not differ significantly between the ECRS and CONT groups, but showed associations with hippocampal glial markers and *Il-6* expression. These taxa may therefore reflect inter-individual variability in hippocampal neuroimmune status rather than disease-specific enrichment.

Together, these findings indicate a link between nasal microbiota compositions and hippocampal neuroimmune alterations in the ECRS model. These findings may help explain, at least in part, the epidemiological observations linking CRS to an increased risk of depression, anxiety, and cognitive impairment. Although the present study identified a closer association between the nasal microbiota and hippocampal neuroimmune alterations in this ECRS model, accumulating evidence suggests that modulation of the gut microbiota through microbiota-targeted approaches may influence inflammatory diseases via systemic immune regulation. Future studies should determine whether modulation of the nasal microbiota, the gut microbiota, or both can influence neuroimmune alterations and disease progression in CRS/ECRS.

### 4.6 Limitation

In the present study, only adult male mice were used; therefore, age- and sex-dependent differences could not be assessed. In addition, the SPF environment in which experimental animals are maintained differs substantially from the complex microbial environments encountered by humans, which may limit the direct extrapolation of our findings to human patients. Furthermore, the present study is based on association analyses, and thus does not allow inference of causal relationships between nasal microbiota alterations and hippocampal neuroimmune changes. Future studies incorporating female mice, animals of different ages, and microbiota-targeted mechanistic interventions will be necessary to clarify whether specific bacterial taxa causally contribute to hippocampal neuroimmune alterations in ECRS.

## 5 Conclusion

In conclusion, the ECRS mouse model demonstrated dysbiosis of the nasal and gut microbiota accompanied by hippocampal neuroimmune alterations. Several nasal bacterial taxa were associated with hippocampal glial and inflammatory gene expression. These findings support the concept that the nasal microbiota composition is linked to hippocampal neuroimmune alterations in ECRS.

## Data Availability

The raw sequencing data generated in this study have been deposited in the DDBJ Sequence Read Archive (DRA) under Run accession numbers DRR1088657–DRR1088692. The data are publicly available at https://ddbj.nig.ac.jp/search/entry/bioproject/PRJDB42977/.
