# Supplementary material for: Dysbiosis of nasal microbiota associated with hippocampal neuroimmune alterations in a mouse model of eosinophilic chronic rhinosinusitis
Source: Front Immunol. 2026 Sep 3;17:1811570. doi: 10.3389/fimmu.2026.1811570 (PMC13581562; doi:10.3389/fimmu.2026.1811570)
Supplement: Supplementary file 1 [file DataSheet1.pdf]

Supplementary Figure S1

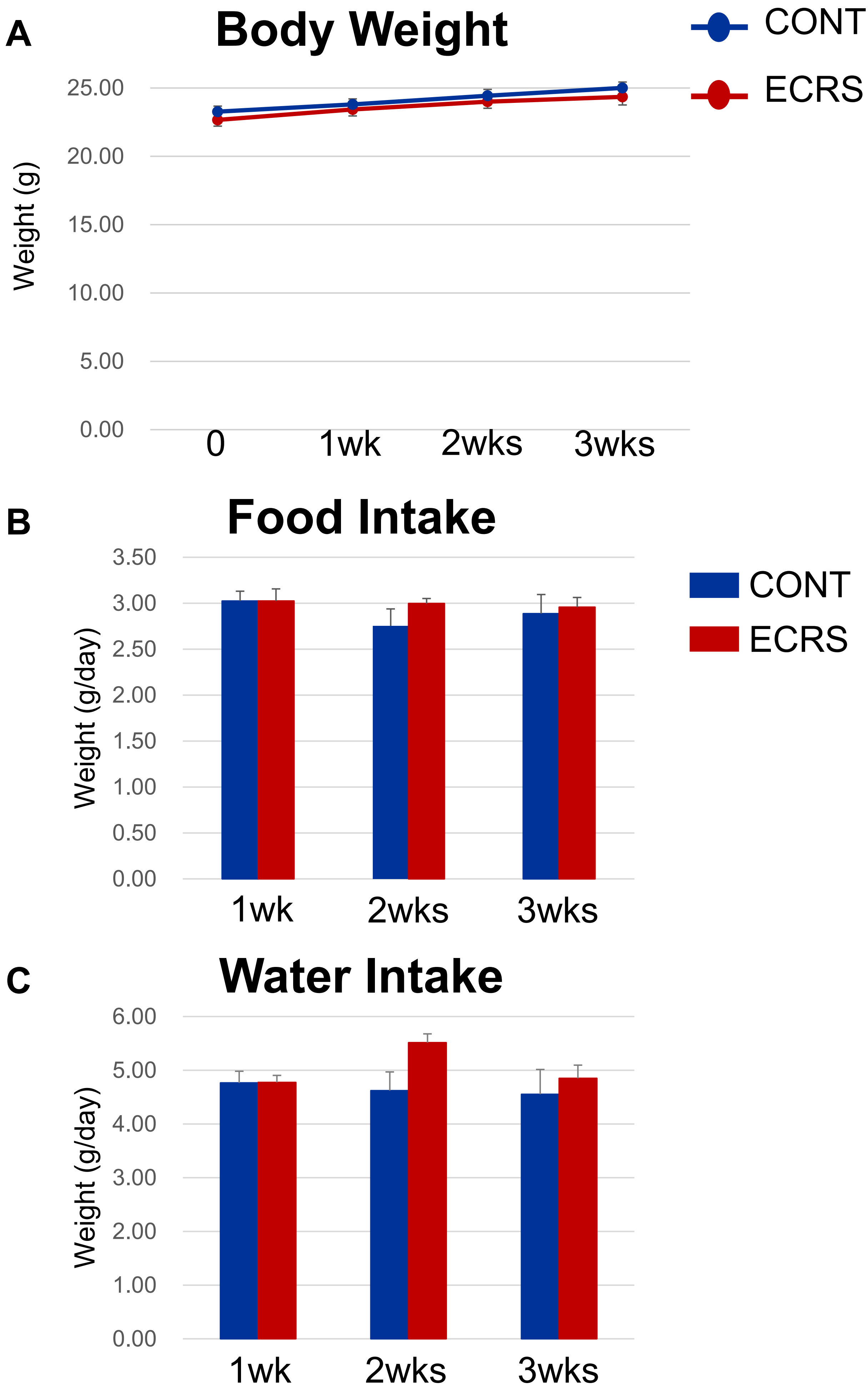

Supplementary Figure S1. Physiological changes during ECRS induction.

(A) Changes in body weight over the 3-week experimental period. No significant differences were observed between the ECRS and control (CONT) groups. CONT, n = 10; ECRS, n = 11.

(B) Food intake (g/day) over the 3-week period. No significant differences were observed between the ECRS and CONT groups. CONT, n = 3; ECRS, n = 3.

(C) Water intake (g/day) over the 3-week period. No significant differences were observed between the ECRS and CONT groups. CONT, n = 3; ECRS, n = 3.

Supplementary Figure S2

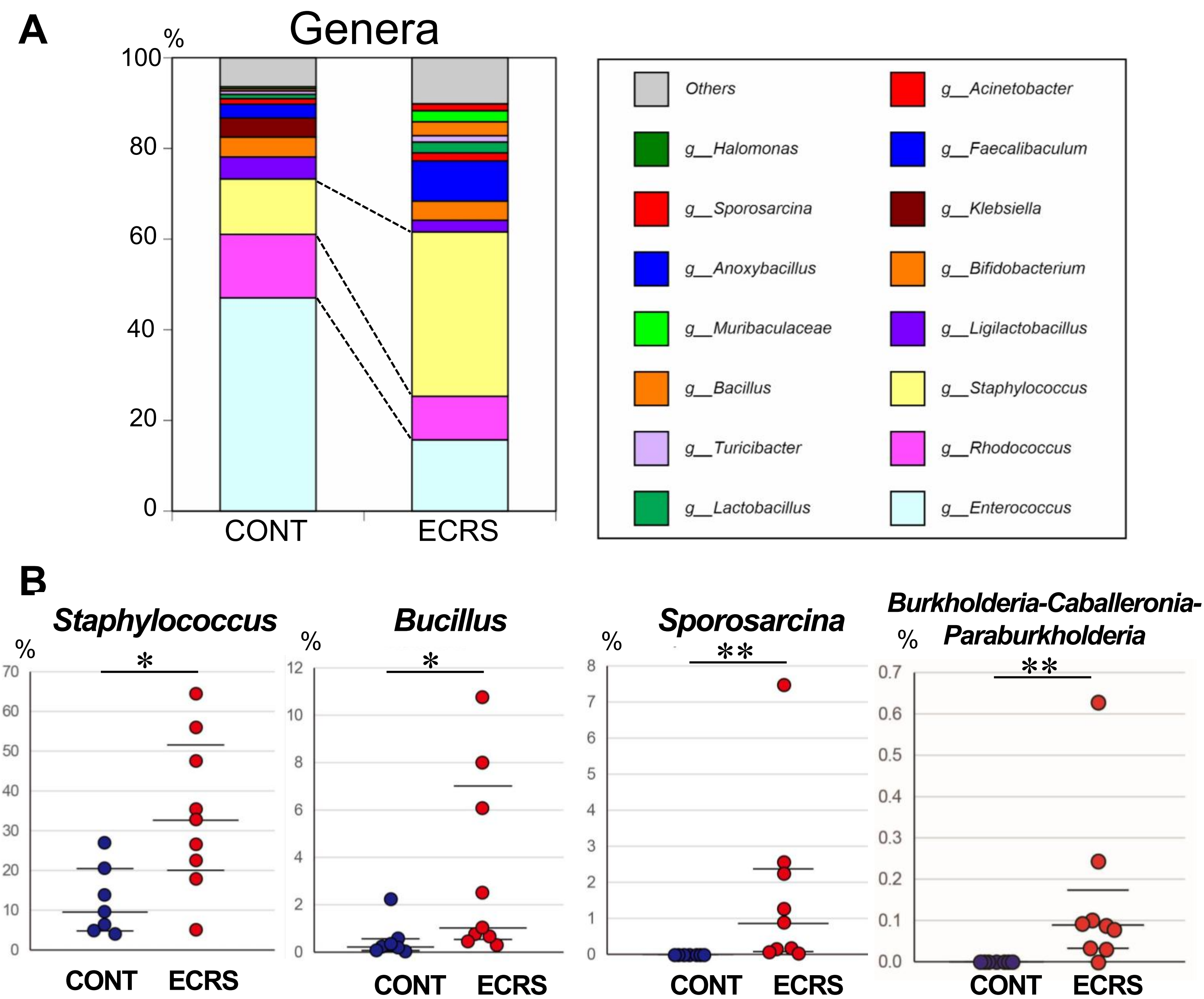

Supplementary Figure S2. Alterations in nasal microbiota composition at the genus level in the ECRS group.

(A) Nasal microbial community composition at the genus level. (B) Genera showing significantly altered relative abundance in the ECRS group compared with the control (CONT) group. The relative abundances of *Staphylococcus*, *Bacillus*, *Sporosarcina*, and *Burkholderia-Caballeronia-Paraburkholderia* were increased in the ECRS group, as determined by the Mann-Whitney U test. * $p < 0.05$, ** $p < 0.01$. CONT, $n = 7$; ECRS, $n = 9$.

Supplementary Figure S3

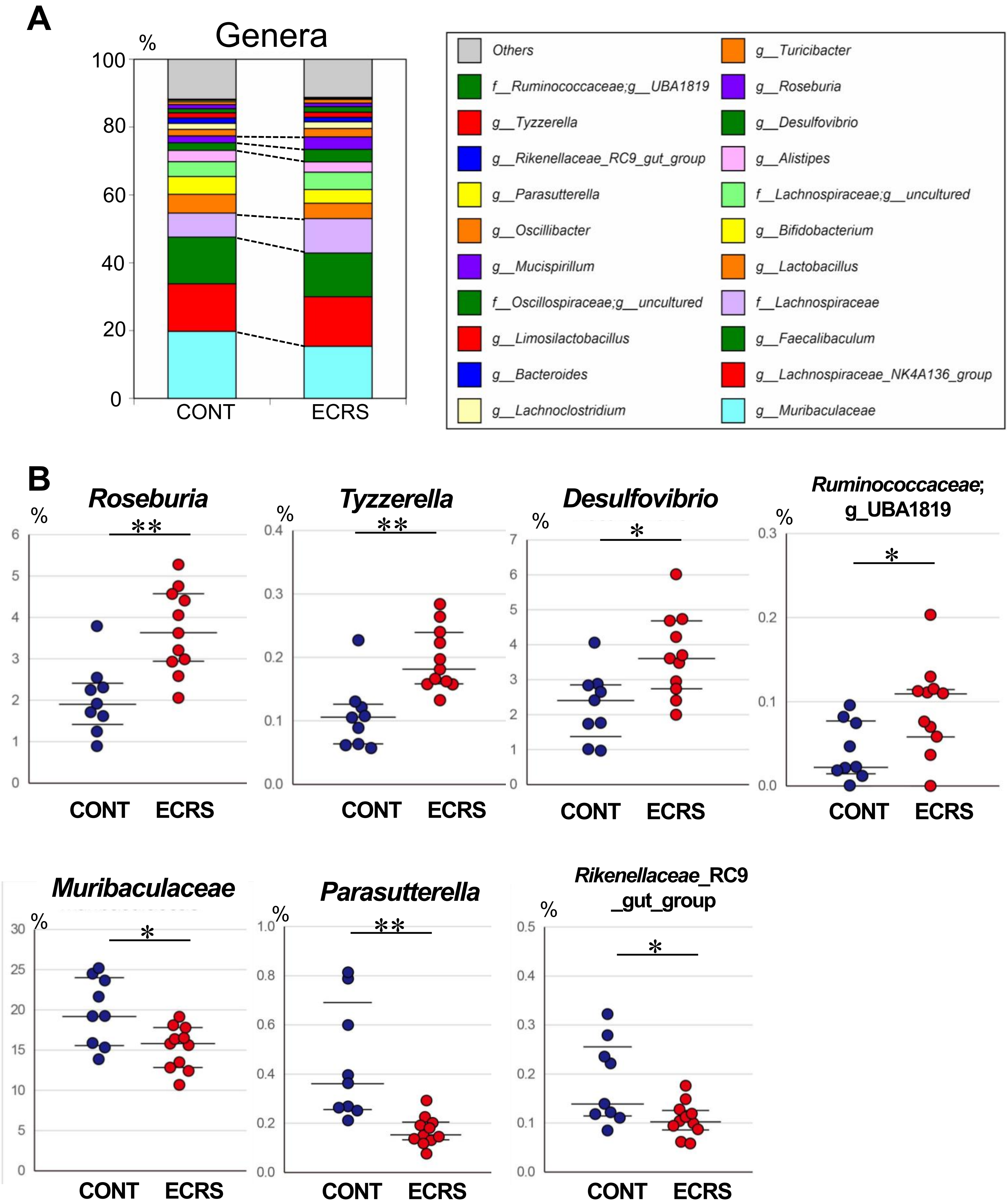

Supplementary Figure S3. Alterations in gut microbiota composition at the genus level in the ECRS group.

(A) Gut microbial community composition at the genus level. (B) Genera showing significantly altered relative abundance in the ECRS group compared with the control (CONT) group. The relative abundances of *Roseburia*, *Tyzzerella*, *Desulfovibrio*, and *Ruminococcaceae*_UBA1819 were increased, whereas those of *Muribaculaceae*, *Parasutterella*, and *Rikenellaceae*_RC9_gut_group were decreased in the ECRS group, as determined by the Mann-Whitney U test. * $p < 0.05$, ** $p < 0.01$. CONT, n = 9; ECRS, n = 11.

Supplementary Figure S4

Olfactory bulb x gut microbiota

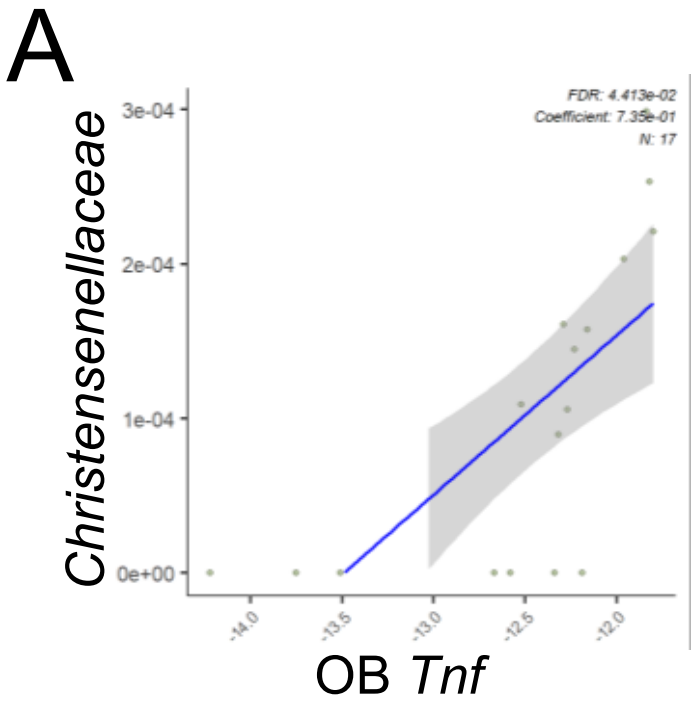

Hippocampus x Nasal microbiota

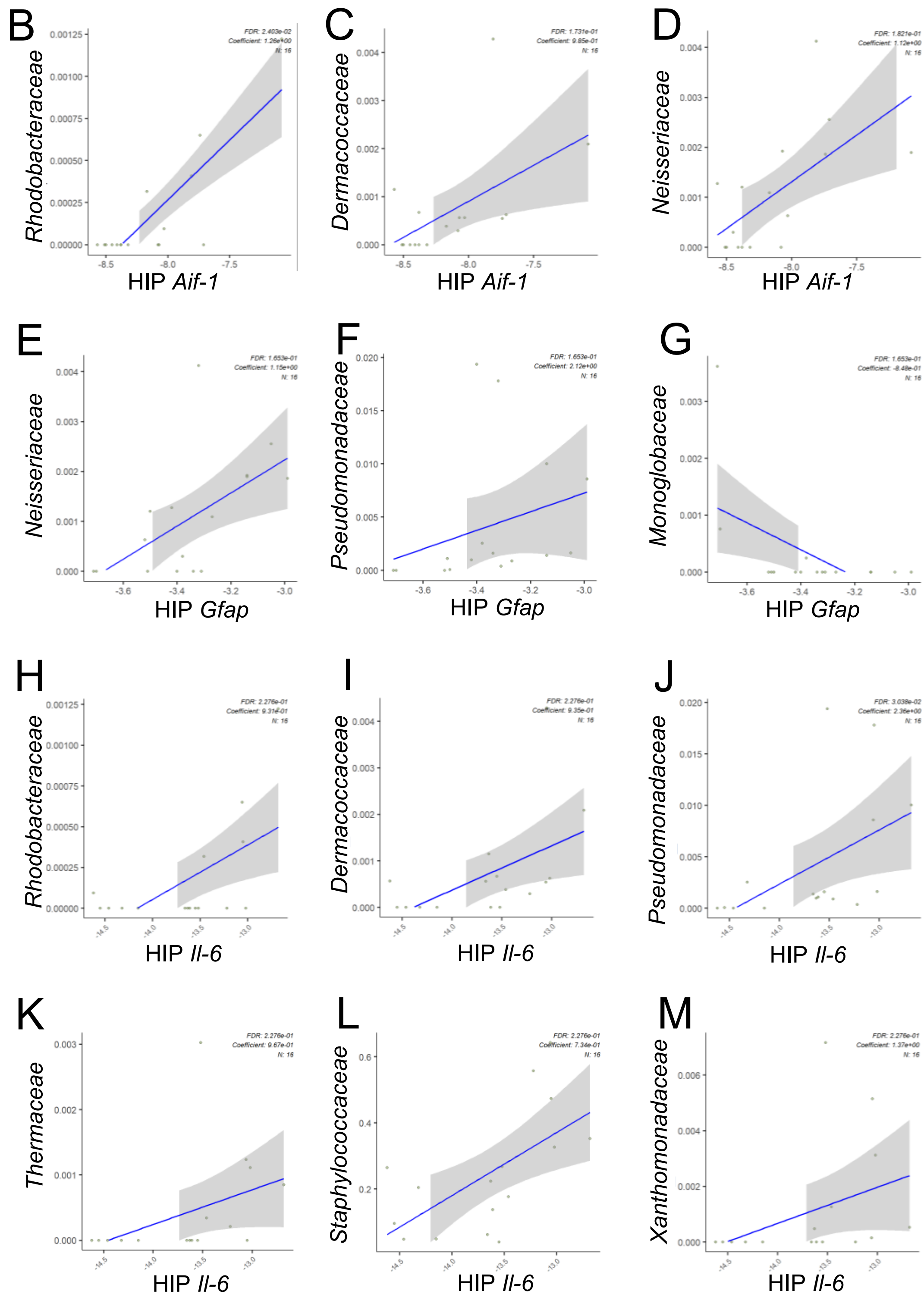

Supplementary Figure S4. Associations between brain gene expression and the nasal and gut microbiota identified by MaAsLin2.

Scatter plots showing significant associations identified by MaAsLin2 between brain gene expression and microbial taxa.

(A) Significant positive association between *TNF* expression in the olfactory bulb and the relative abundance of gut *Christensenellaceae*.

(B-D) Significant positive associations between hippocampal *Aif-1* expression and nasal microbial taxa.

(E-G) Significant positive associations and one significant negative association between hippocampal *Gfap* expression and nasal microbial taxa.

(H-M) Significant positive associations between hippocampal *Il-6* expression and nasal microbial taxa.

Supplementary Figure S5

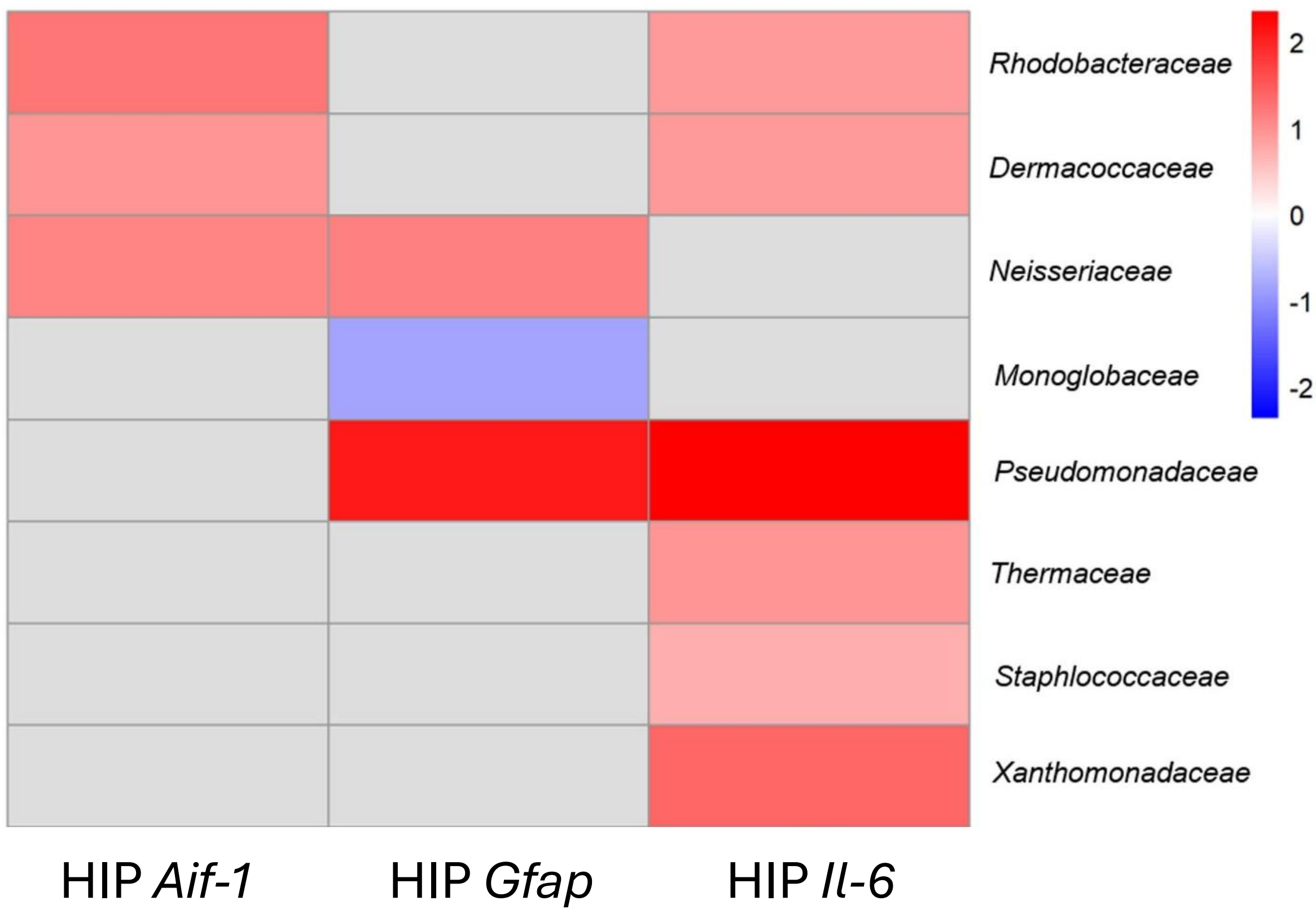

Supplementary Figure S5. Associations between hippocampal gene expression and nasal microbiota.

Heatmap showing significant associations identified by MaAsLin2 between hippocampal gene expression and nasal bacterial taxa. Hippocampal *Aif-1* expression was positively associated with the families *Rhodobacteraceae*, *Dermacoccaceae*, and *Neisseriaceae*. Hippocampal *Gfap* expression was positively associated with the families *Neisseriaceae* and *Pseudomonadaceae,* and negatively associated with the family *Monoglobaceae*. Hippocampal *Il-6* expression was positively associated with the families *Rhodobacteraceae*, *Dermacoccaceae*, *Pseudomonadaceae*, *Thermaceae*, *Staphylococcaceae*, and *Xanthomonadaceae*.

Supplementary Table S1

A

Nasal microbiota

Taxa	Relative abundance		P value
	CONT (%)	ECRS (%)	
Staphylococcaceae	12.22	34.07	P=0.0299
Bacillaceae	0.60	3.49	P=0.0299
Planococcaceae	0.00	1.62	P=0.0091
Burkholderiaceae	0.00	0.15	P=0.0091
Ruminococcaceae	0.00	0.14	P=0.0826
Halomonadaceae	0.00	0.04	P=0.0826
Beijerinckiaceae	0.06	0.22	P=0.0826
Dermacoccaceae	0.02	0.11	P=0.0615
Rhodobacteraceae	0.00	0.03	P=0.0826
Propionibacteriaceae	0.08	0.12	P=0.0558
Enterococcaceae	47.11	14.12	P=0.0558

B

Gut microbiota

Taxa	Relative abundance		P value
	CONT (%)	ECRS (%)	
Lachnospiraceae	33.64	39.84	P=0.0497
Desulfovibrionaceae	2.52	3.90	P=0.0230
Muribaculaceae	19.84	15.42	P=0.0497
Sutterellaceae	0.44	0.17	P=0.0029

Supplementary Table S1. Differential abundance of microbial families.

(A) Table showing the relative abundances of nasal microbial families that differed significantly between the ECRS and control (CONT) groups, together with the results of the Mann-Whitney U test. CONT, n = 7; ECRS, n = 9.

(B) Table showing the relative abundances of gut microbial families that differed significantly between the ECRS and CONT groups, together with the results of the Mann-Whitney U test. CONT, n = 9; ECRS, n = 11.

Relative abundances are presented as mean percentages. Red indicates $p < 0.05$, and blue indicates $0.05 \leq p < 0.10$.

Supplementary Table S2

Pathway ID	Pathway Annotation	Coefficient	P value	Q value	Detection	Interpretation
PWY-4984	Urea cycle	1.155	7.11e-06	0.003	16/16	Higher predicted abundance in ECRS-N
PWY-5304	Superpathway of sulfur oxidation	1.909	0.000115	0.021	16/16	Higher predicted abundance in ECRS-N
PWY-1861	Formaldehyde assimilation II (assimilatory RuMP cycle)	1.215	0.000373	0.035	16/16	Higher predicted abundance in ECRS-N
RUMP-PWY	Formaldehyde oxidation I	1.165	0.000341	0.035	16/16	Higher predicted abundance in ECRS-N
PWY-3001	Superpathway of L-isoleucine biosynthesis I	0.282	0.00139	0.089	16/16	Higher predicted abundance in ECRS-N
PWY-7295	L-arabinose degradation IV	2.007	0.00143	0.089	10/16	Higher predicted abundance in ECRS-N
PWY-5265	Peptidoglycan biosynthesis II (staphylococci)	0.642	0.00171	0.091	16/16	Higher predicted abundance in ECRS-N

Supplementary Table S2. Predicted microbial functions.

The suffix “N” denotes the nasal microbiota. The CONT-N group was used as the reference group. Positive coefficients therefore indicate higher predicted pathway abundance in the ECRS-N. Pathways with $q < 0.05$ are considered the primary findings, whereas those with $0.05 \leq q < 0.10$ are considered exploratory. PICRUSt2 estimates functional potential from 16S rRNA gene data and does not directly measure pathway activity or metabolite production.

Supplementary Table S3

Nasal microbiota

Bacterial taxa	Microbiologial characteristics	Alteration in ECRS		Association identified by MaAsLin2
		P value	LEfSe	
Staphylococcaceae	Gram-positive bacteria commonly found on the skin and mucosal surfaces of humans and animals.	↑	↑	Hip *Il-6*
Bacillaceae	Gram-positive, spore-forming bacteria that are widely distributed in soil and other environmental habitats.	↑	↑	
Planococcaceae	Gram-positive bacteria commonly found in soil, water, and other environmental niches.	↑	↑	
Burkholderiaceae	Gram-negative bacteria that inhabit soil, water, plants, and animal-associated environments.	↑	↑	
Ruminococcaceae	Anaerobic Gram-positive bacteria that are abundant members of the mammalian gut microbiota.	(↑)	↑	
Halomonadaceae	Halophilic or halotolerant Gram-negative bacteria typically found in saline environments.	(↑)	↑	
Beijerinckiaceae	Gram-negative bacteria commonly found in soil and freshwater, including several methylotrophic species.	(↑)		
Dermacoccaceae	Gram-positive Actinobacteria that are frequently isolated from the skin and environmental sources.	(↑)		Hip *Aif-1* , Hip *Il-6*
Rhodobacteraceae	Gram-negative bacteria widely distributed in marine and freshwater environments.	(↑)	↑	Hip *Aif-1* , Hip *Il-6*
Propionibacteriaceae	Gram-positive bacteria that commonly colonize the skin and sebaceous glands of humans.	(↑)	↑	
Enterococcaceae	Gram-positive lactic acid bacteria that inhabit the gastrointestinal tract of humans and animals.	(↓)		
Neisseriaceae	Gram-negative bacteria that colonize the mucosal surfaces of humans and other animals.			Hip *Aif-1* , Hip *Gfap*
Monoglobaceae	A recently described family of anaerobic bacteria that has primarily been identified in the mammalian intestinal microbiota.			Hip *Gfap* (-)
Pseudomonadaceae	Gram-negative bacteria that are widely distributed in soil, water, and plant-associated environments.			Hip *Gfap* , Hip *Il-6*
Thermaceae	Thermophilic Gram-negative bacteria that thrive in high-temperature environments such as hot springs.			Hip *Il-6*
Xanthomonadaceae	Gram-negative bacteria that includes species inhabiting plants, soil, water, and clinical environments.			Hip *Il-6*

Gut microbiota

Taxa	Description	Alteration in ECRS		Association identified by MaAslin2
		P value	LEfSe	
Lachnospiraceae	Anaerobic Gram-positive bacteria that are common members of the mammalian gut microbiota.	↑	↑	
Desulfovibrionaceae	Anaerobic Gram-negative bacteria that reduce sulfate and are commonly found in the gastrointestinal tract.	↑	↑	
Muribaculaceae	Anaerobic bacteria that are abundant in the gut microbiota of rodents, particularly mice.	↓	↓	
Sutterellaceae	Gram-negative bacteria that commonly inhabit the intestinal tract of humans and other mammals.	↓	↓	
Christensenellaceae	Aanaerobic Gram-positive bacteria that are common members of the mammalian gut microbiota and are frequently associated with a healthy intestinal microbial community.			OB *Tnf*

Supplementary Table S3. Summary of nasal and gut bacterial families identified across the Mann-Whitney U test, LEfSe, and MaAsLin2 analyses.

(A) Summary table of nasal microbial families, including their relative abundances, biological characteristics, and the results of the Mann-Whitney U test, LEfSe, and MaAsLin2 analyses.

(B) Summary table of gut microbial families, including their relative abundances, biological characteristics, and the results of the Mann-Whitney U test, LEfSe, and MaAsLin2 analyses.

↑, significantly increased in ECRS compared with CONT (Mann-Whitney U test or LEfSe)

(↑), a trend toward an increase in ECRS compared with CONT (Mann Whitney U test)

↓, significantly decreased in ECRS compared with CONT (Mann-Whitney U test or LEfSe)

Hip, hippocampus; OB, olfactory bulb
